# Adaptation of the GoldenBraid modular cloning system and creation of a toolkit for the expression of heterologous proteins in yeast mitochondria

**DOI:** 10.1186/s12896-017-0393-y

**Published:** 2017-11-13

**Authors:** Ana Pérez-González, Ryan Kniewel, Marcel Veldhuizen, Hemant K. Verma, Mónica Navarro-Rodríguez, Luis M. Rubio, Elena Caro

**Affiliations:** 1 Centro de Biotecnología y Genómica de Plantas, Universidad Politécnica de Madrid (UPM) - Instituto Nacional de Investigación y Tecnología Agraria y Alimentaria (INIA), Campus Montegancedo UPM, 28223 Pozuelo de Alarcón, Madrid Spain; 2 Present Address: Department of Environmental Biology, Centro de Investigaciones Biológicas, Consejo Superior de Investigaciones Científicas (CIB-CSIC), 28040 Madrid, Spain; 3 Present Address: Mankind Research Centre, IMT Manesar, Gurgaon, Haryana 122050 India

**Keywords:** Synthetic biology, GoldenBraid, *Saccharomyces cerevisiae*, Mitochondria

## Abstract

**Background:**

There is a need for the development of synthetic biology methods and tools to facilitate rapid and efficient engineering of yeast that accommodates the needs of specific biotechnology projects. In particular, the manipulation of the mitochondrial proteome has interesting potential applications due to its compartmentalized nature. One of these advantages resides in the fact that metalation occurs after protein import into mitochondria, which contains pools of iron, zinc, copper and manganese ions that can be utilized in recombinant metalloprotein metalation reactions. Another advantage is that mitochondria are suitable organelles to host oxygen sensitive proteins as a low oxygen environment is created within the matrix during cellular respiration.

**Results:**

Here we describe the adaptation of a modular cloning system, GoldenBraid2.0, for the integration of assembled transcriptional units into two different sites of the yeast genome, yielding a high expression level*.* We have also generated a toolkit comprising various promoters, terminators and selection markers that facilitate the generation of multigenic constructs and allow the reconstruction of biosynthetic pathways within *Saccharomyces cerevisiae*. To facilitate the specific expression of recombinant proteins within the mitochondrial matrix, we have also included in the toolkit an array of mitochondrial targeting signals and tested their efficiency at different growth conditions. As a proof of concept, we show here the integration and expression of 14 bacterial nitrogen fixation (*nif*) genes, some of which are known to require specific metallocluster cofactors that contribute to their stability yet make these proteins highly sensitive to oxygen. For one of these genes, *nifU*, we show that optimal production of this protein is achieved through the use of the Su9 mitochondrial targeting pre-sequence and glycerol as a carbon source to sustain aerobic respiration.

**Conclusions:**

We present here an adapted GoldenBraid2.0 system for modular cloning, genome integration and expression of recombinant proteins in yeast. We have produced a toolkit that includes inducible and constitutive promoters, mitochondrial targeting signals, terminators and selection markers to guarantee versatility in the design of recombinant transcriptional units. By testing the efficiency of the system with nitrogenase Nif proteins and different mitochondrial targeting pre-sequences and growth conditions, we have paved the way for future studies addressing the expression of heterologous proteins in yeast mitochondria.

**Electronic supplementary material:**

The online version of this article (10.1186/s12896-017-0393-y) contains supplementary material, which is available to authorized users.

## Background

*Saccharomyces cerevisiae* is a single celled yeast commonly used for biotechnological and synthetic biology applications that has the ability to either ferment or respire to grow depending on the available carbon source(s). Using yeast as a chassis for biotechnology is impactful as it serves as a starting point for more complicated engineering projects in higher eukaryotes that share many of its metabolic and cellular characteristics. Several modular cloning methods exist for engineering yeast, such as MoClo-YTK [1] and yeast GoldenGate (yGG) [2]. There is need, however, for the development of novel synthetic biology tools to accommodate the necessities of specific projects.

Mitochondria play a fundamental role in ATP generation through cellular respiration and execute critical biochemical functions for the synthesis of fatty acids, amino acids and nucleotides. Many proteins containing metallocluster cofactors are required for these essential physiological and biochemical processes. Zinc, iron, copper and manganese metal cofactors are required for normal mitochondrial function and are relatively abundant within the matrix [3]. One strategy to benefit from the manipulation of yeast mitochondria is the targeting of recombinant metalloproteins into the organelle. Once imported, these metalloproteins can be properly folded and incorporate their required metal cofactors, taking advantage of the specific metal availability within the matrix.

Another strategy consists of benefitting from the respiratory activity of mitochondria. Respiration is used in many diazotrophic bacteria during the synthesis of enzymes involved in nitrogen fixation. This is hypothesized to decrease oxygen tension on the oxygen-sensitive nitrogenase enzymes─the so-called “respiratory protection” of nitrogenase hypothesis [3]. According to this hypothesis, the respiratory electron transport system performs an O_2_-scavenging function preventing the diffusion of O_2_ into the cells, keeping the interior of the cells anoxic even with high ambient O_2_ concentrations. In fact, the convenience of using yeast mitochondria to host nitrogenase components was recently shown. There have been previous efforts to express *nif* genes in eukaryotic organisms such as *S. cerevisiae* [4–8], *Chlamydomonas reinhardtii* [9] and even plants [10–12]. Frequently, these efforts resulted in producing inactive or nearly inactive nitrogenase enzymes. However, the expression of *nifH* and *nifM* in *S. cerevisiae* mitochondria using mitochondrial targeting was successful in obtaining active enzymes under aerobic growth conditions [13].

In this report we describe the adaptation of a modular cloning method originally designed for plants, GoldenBraid2.0 [14], for *S. cerevisiae* genome integration and gene expression. It is a modular cloning system that relies on the use of type IIS restriction enzymes for DNA assembly and that generates fully exchangeable genetic elements for multigene engineering, comparable with existing MoClo-YTG and yGG strategies.

We have also generated a synthetic biology toolkit that includes promoters of various strengths, an array of mitochondrial targeting signals, terminators and selection markers to use in the reconstruction of biosynthetic pathways in the mitochondrial matrix of *S. cerevisiae*. This new method has been tested for several Nif proteins and different growth conditions were compared to obtain optimal results.

## Methods

### Generation of GoldenBraid plasmids adapted for yeast genome integration

To generate new integration constructs compatible with the GoldenBraid2.0 standard, the backbone of GoldenBraid (GB) vectors pDGB2α1 and pDGB2Ω1 were amplified using primers 1826/1827 and 1827/1828, respectively, and new synthetic GoldenBraid cloning cassettes adapted for *S. cerevisiae* genome integration were ligated within. These *S. cerevisiae*-specific synthetic cassettes were synthetized by Genscript and consisted of recombination arms of homology for integration at the *YPRCΔ15* or *YORWΔ22* solo long terminal repeat (LTR) loci described in [14]. Integration at these loci conferred a high level of expression of a reporter gene using two different promoters (*TEF1*p and *ACT1*p). These integrative arms of homology flanked the regular GB module (BsmBI-BsaI-*lacZ*-BsaI-BsmBI for α1 or BsaI-BsmBI-*lacZ*-BsmBI-BsaI for Ω1 vectors [15]) and the entire cassette was flanked by I-SceI restriction sites. This resulted in the construction of 4 plasmids, YPRCΔ15α1, YPRCΔ15Ω1, YORWΔ22α1 and YORWΔ22Ω1. Subsequently, the α1 vectors were mutagenized to become α2 and the Ω1 vectors were mutagenized to become Ω2, generating 8 total plasmids (Genscript).

### Molecular biology enzymes

Enzymes used for molecular biology were the following: restriction enzymes BsmBI/Esp3I (Fermentas), BsaI and BtgZI (NEB), T4 DNA ligase (Promega), Phusion DNA Polymerase (Agilent) and KAPA2G (KAPA Biosystems).

### Domestication of parts

GB parts were amplified by PCR from plasmid or genomic DNA templates with primers designed by using the Domesticator tool of www.GBcloning.org as described in Additional file 1: Table S1 and Additional file 2: Table S2. Domestication of GB parts is the process by which the internal restriction sites BsmBI/Esp3I, BsaI and BtgZI are removed and appropriate 4 nucleotide flanking overhangs are added to provide specificity to the part type. The sequence of each part is listed in Additional file 3: Figure S1. The DNA sequence encoding the 8xHis tag and the codon-optimized versions of the *nif* genes were synthetized by Genscript or Proteogenix, as described in Additional file 1: Table S1. The protocol used for the domestication reactions consists of adding 40 ng of each DNA patch, 75 ng of pUPD plasmid, 10 units of BsmBI, 3 units of T4 DNA ligase and 1 μL of 10× ligase buffer in a final volume of 10 μL. Reactions were carried out in a thermocycler, 25 cycles of 37 °C 2 min, 16 °C 5 min [15].

### Cloning into destination plasmids

After domestication of all GB parts in the universal domesticator plasmid, pUPD, the desired transcriptional units (TUs) were generated in the α1 destination plasmids. The *kanMX* (G418R) or the *hphMX* (HygroR) resistance selection markers were digested from pUPD and subcloned into the α2 plasmids. α1-TUs and α2-selection markers were then combined into Ω1 plasmids. The protocol for the TU assembly reaction in α destination plasmids consists on adding 75 ng of each part, 75 ng of the α destination plasmid, 10 units of BsaI, 3 units of T4 DNA ligase and 1 μL of 10× ligase buffer in a final volume of 10 μL. Reactions were carried out in a thermocycler, 25 cycles of 37 °C 2 min, 16 °C 5 min [15]. The protocol for the piling up of the TUs cloned in the two α plasmids into an Ω plasmid consists on adding 75 ng of each α plasmid, 75 ng of the Ω plasmid, 10 units of BsmBI, 3 units of T4 DNA ligase and 1 μL of 10× ligase buffer in a final volume of 10 μL. Reactions were carried out in a thermocycler, 25 cycles of 37 °C 2 min, 16 °C 5 min [15].

Verification of the final constructs was performed by Sanger sequencing using a promoter forward primer, a terminator reverse primer and an internal primer when needed for longer length parts.

### Strains and growth media

The *S. cerevisiae* strains W303–1A (*ura3–1; trp1–1; leu2–3112; his3–11; ade2–1; can1–100*) (ATCC 208352) and CEN.PK2–1D (*ura3–52; trp1–289; leu2–3112; his3Δ 1; MAL2-8C; SUC2*) were used as indicated in the text and figures. Cells were grown in YPAU media (1% yeast extract (Conda), 2% bactopeptone (Pronadisa), 0.2 mg/ml adenine sulfate (Formedium) and 0.27 mg/ml uracil (Amresco)) with 2% glucose (Sigma), 2% galactose (Formedium) or 3% glycerol (GPR Rectapur VWR) as described in each case.

### *S. cerevisiae* Transformation

The final plasmids containing the GB-assembled TU constructs were digested with I-SceI (NEB) to generate a linear DNA fragment that was used for *S. cerevisiae* transformation and genome integration, as described in reference [16].

G418 (200 μg/ml, Santa Cruz Biotechnology) and Hygromycin B (200μg/ml, Formedium) were used for selection. Transformants were purified by restreaking on YPAUD plates containing 2% glucose and the appropriate antibiotic, and only colonies growing with normal morphology were selected for further experimentation.

Verification of genomic integration was performed for each strain by extracting genomic DNA using the Bust n’ Grab Genomic DNA Isolation Protocol [17] and by carrying out PCR reactions for the *YPRC∆15* or *YORW∆22* integration loci using primers specific to sequences flanking the integration arms of homology together with internal primers specific to each construct. To confirm integration at the *YPRC∆15* locus, we used the 5′ flanking forward primer 1833 and a reverse primer internal to the promoter of the *nif* transcription unit. To confirm *YPRC∆15* 3′ integration, we used a *kanMX* internal forward primer 1551 and 3′ flanking reverse primer 1836. To confirm *YORW∆22* 5′ integration, we used 5′ flanking forward primer 1829 and a reverse primer internal to the promoter of the *nif* transcription unit. To confirm *YORW∆22* 3′ integration, we used a *kanMX* or *hphMX* internal forward primer 1551 or 1556 and 3′ flanking reverse primer 1832. Primer sequences are listed in Additional file 2: Table S2.

### Preparation of yeast extracts and immunoblotting

Cell pellets corresponding to OD_600_ 5–10 were protein extracted using the alkali extraction protocol described in [18], and samples corresponding to OD_600_ 0.5–1 were analyzed by SDS-PAGE and western blotting (WB). Monoclonal mouse anti-His primary antibody (Sigma H1029-2ML, 1:5000 dilution) and anti-mouse-HRP secondary antibody (Santa Cruz Biotechnology sc-2060, 1:10,000 to 1:20,000 dilution), or polyclonal rabbit anti-NifU ([13], 1:5000 dilution), polyclonal rabbit anti-NifS ([13], 1:200 dilution), polyclonal rabbit anti-NifM (1:4000 dilution), and anti-rabbit-HRP secondary antibody (Sigma A0545-1ML, 1:10,000 dilution) were used.

After the immunodetection of proteins, polyvinylidene fluoride (PVDF) membranes were stained with Coomassie to determine the total protein loaded and the blotting efficiency, as described in [19].

Representative expression results are shown throughout the manuscript. For every figure, at least two transformant colonies were analyzed for each strain and the results were obtained from at least two biologically independent experiments.

### Preparation of mitochondria enriched extracts

The preparation of a crude mitochondrial fraction was performed following the protocol described in [20] with 5 g of cells collected from cultures grown in YPAU media with 2% glucose to a final OD_600_ of 4–6.

Western blots were carried out using polyclonal rabbit anti-NifU ([13], 1:5000 dilution), monoclonal mouse anti-HSP60 (Novus biologicals, NBP2–34671H, 1:2000 dilution), monoclonal rat anti-tubulin (Santa Cruz Biotechnology sc-69,971, 1:1000 dilution), anti-rabbit-HRP secondary antibody (Sigma A0545-1ML, 1:10,000 dilution), anti-mouse-HRP secondary antibody (Santa Cruz Biotechnology, sc-2060, 1:20,000 dilution) and anti-rat-HRP secondary antibody (Amersham NA 935, 1:10,000 dilution).

### Glucose determination in culture media

Glucose presence in the media was estimated using a Glucocard G+ meter sensor.

## Results

### Adaptation of the GoldenBraid system for yeast

**Fig. 1:**
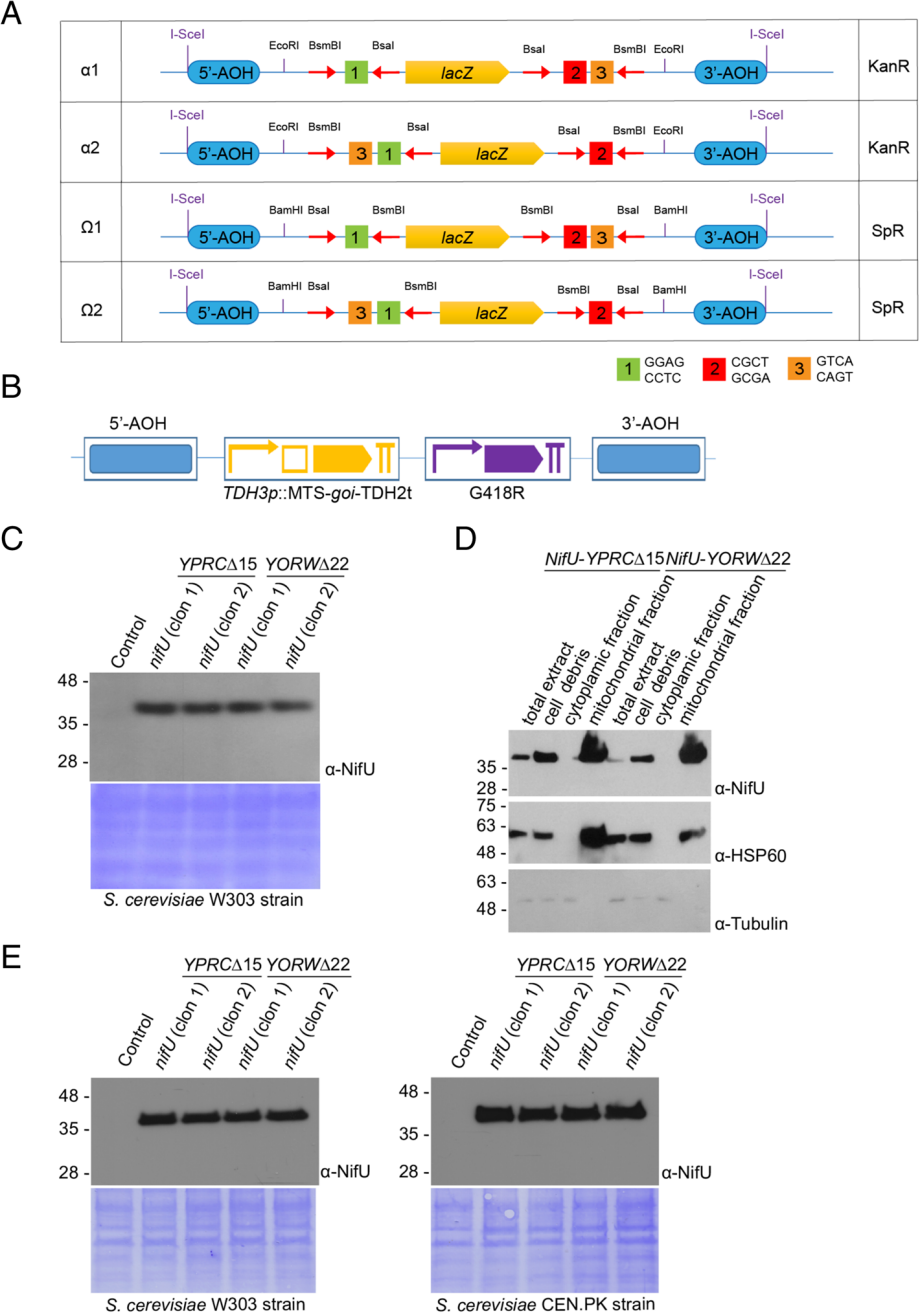
Adaptation of GoldenBraid2.0 system for integration of constructs in *S. cerevisiae* genome. **a** Schematic representation of the GoldenBraid2.0 plasmids adapted for integration of constructs into the genome of *S. cerevisiae*. **b** Diagram of the two transcriptional units constructs used in (**c**-**e**) (AOH, arms of homology; *goi*, gene of interest; G418R, *kanMX* G418 resistance cassette, for (**c**-**d**), MTS: Su9, for (**e**), MTS: MAM33). **c** Comparison of NifU expression in two transformants after integration in the *YPRCΔ15* and *YORWΔ22* loci of *S. cerevisiae* strain W303. Anti-NifU western blot of alkaline whole cell extracts from saturated cultures containing glucose as the carbon source were used. Control refers to non-transformed cells. The lower panel shows the corresponding membrane Coomassie stained as a loading control. **d** Mitochondrial fractionation with lanes corresponding to total cell extract, pelleted cell debris after centrifugation, cytoplasmic and mitochondrial-enriched fractions from cells in (**c**) (transformant colony one). Anti-NifU WB is shown in the upper panel, anti-HSP60 WB (mitochondrial matrix marker) is the middle panel and anti-tubulin WB (cytoplasmic marker) is the lower panel. **e** Comparison of NifU expression in two transformants after integration into the *YPRCΔ15* and *YORWΔ22* sites of W303 and CEN.PK *S. cerevisiae* strains. Saturated cultures containing glucose as the carbon source were used. Control refers to non-transformed cells. The lower panel shows the corresponding membrane Coomassie stained as a loading control

We sought to develop a reliable and modular system to integrate TUs of heterologous genes into the genome of *S. cerevisiae*. To meet this end, we adopted the GoldenBraid2.0 cloning system, a system that was originally developed for plants. GoldenBraid modular cloning plasmids were adapted to integrate by homologous recombination at two different positions of the *S. cerevisiae* chromosome that have been validated as suitable for heterologous gene expression: *YPRCΔ15* in chromosome XVI and *YORWΔ22* in chromosome XV [14] (Fig. 1a). Both integration sites are solo long terminal repeats (solo LTRs) and are distal to neighboring coding genetic features, with the nearest being a tRNA gene 831 bp downstream of *YPRCΔ15* and an open reading frame 719 bp upstream of *YORWΔ22.* The adaptation of the GoldenBraid plasmids consisted of a modification to the acceptor plasmids to include arms of homology that allow recombination into these specific locations in the yeast genome, but does not otherwise alter the GoldenBraid cloning mechanism.

As a proof of principle to test the ability to successfully assemble TUs for expression in yeast, constructs were generated containing two TUs. The first TU contained a nitrogenase-specific [Fe–S] cluster biosynthetic protein *nifU* from *A. vinelandii* targeted to the mitochondrial matrix and driven by the constitutive promoter of glyceraldehyde 3-phosphate dehydrogenase (*TDH3*p). NifU was chosen for this proof of concept as it was shown to be stable and accumulate in its functional form in the mitochondrial matrix of *S. cerevisiae* when expressed fused to the Su9 mitochondrial targeting signal (MTS) from *Neurospora crassa* [13]. The second TU is an antibiotic resistance cassette for the selection of integrants. This construct containing two TUs was assembled in both GoldenBraid adapted plasmids containing *YPRCΔ15* or *YORWΔ22* arms of homology and integrated in the two regions of the genome (Fig. 1b). A consistently high expression level of NifU was achieved from two different transformant colonies after integration into either genomic solo LTR region (*nifU-YPRCΔ15* and *nifU-YORWΔ22,* Fig. 1c). Moreover, production of NifU driven by the *TDH3* promoter was similar at both integration sties, as assessed by WB.

Nuclear encoded mitochondrial proteins are synthesized in the cytosol as precursor polypeptides carrying N-terminal MTSs. When transported across the mitochondrial membranes, most proteins are processed from the primary translation product with MTSs being removed by specific processing endopeptidases [21]. NifU proteins were detected as single bands by WB analysis (Fig. 1c), suggesting good expression, mitochondrial targeting and processing. To verify the specific targeting of NifU into the mitochondrial matrix compartment, we prepared mitochondrial extracts from culture-grown cells. NifU was detected in the mitochondria-enriched fraction by WB. The mitochondria-enriched fraction was validated as containing mitochondrial proteins by confirming the presence of the mitochondrial soluble matrix protein HSP60 by WB. In contrast, the mitochondria-enriched fraction was shown to lack the cytoplasmic protein tubulin by anti-tubulin WB (Fig. 1d).

The arm of homology sequences used for integrative recombination perfectly match the corresponding genome sequence of *S. cerevisiae* strain W303. They also have very high homology with the corresponding loci of other strains used for biotechnological applications, such as CEN.PK (95.5% and 99.5% identity for *YPRCΔ15* and *YORWΔ22*, respectively; Additional file 4: Figure S2). We tested the ability of the *nifU-YPRCΔ15* and *nifU-YORWΔ22* constructs to integrate into the genome of CEN.PK yeast and observed similar expression levels of NifU by WB for both constructs as was seen in W303 (Fig. 1e).

### High dynamic range promoter library

**Fig. 2:**
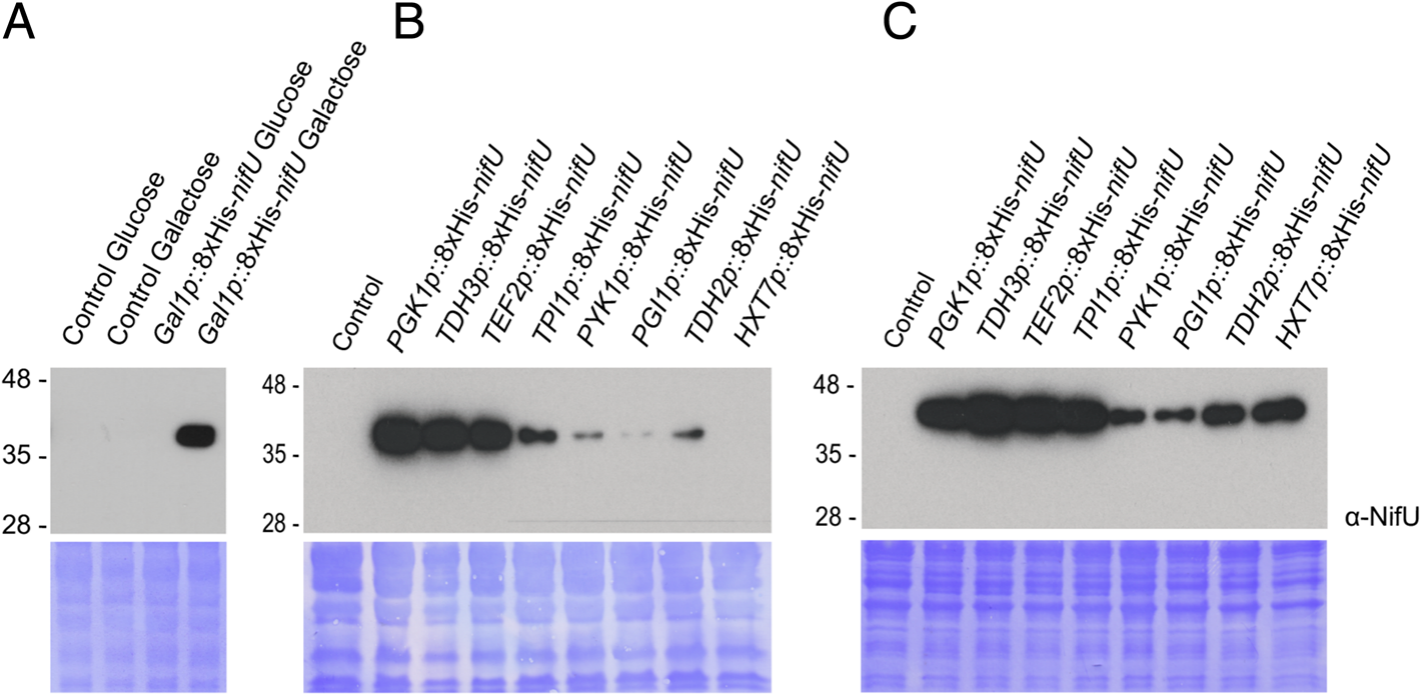
Nine toolkit promoters drive *nifU* expression in different carbon sources. **a** Anti-NifU WB analysis of inducible *nifU* expression driven by the *GAL1*p in exponentially growing cultures containing glucose or galactose as carbon source (CS) as indicated (constructs inserted into *YORWΔ22* locus of strain W303). The lower panel shows the corresponding membrane Coomassie stained as a loading control. **b**-**c** Anti-NifU WB analysis of *nifU* expression driven by different promoters (constructs inserted into *YPRCΔ15* locus of strain W303) in exponentially growing cultures containing glucose (**b**) or glycerol (**c**) as the carbon source. The lower panel shows the corresponding membrane Coomassie stained as a loading control

We aimed for our system to be sufficiently versatile in its design to be useful for a variety of synthetic biology projects. To meet that end, we generated a library of promoters, eight constitutive and one inducible, covering a wide range of expression level and tested them for their ability to drive *nifU* expression (Additional file 5: Figure S3) [22]. The *GAL1*p was used for inducible *nifU* expression after addition of galactose to the growth media (Fig. 2a). The constitutive promoters *PGK1*p, *TDH3*p, *TEF2*p, *TPI1*p, *PYK1p*, *PGI1*p, *TDH2*p *and HXT7*p were tested in cells grown with either glucose (Fig. 2b) or glycerol (Fig. 2c) as the carbon source. Six of these promoters (*PGK1*p, *TDH3*p, *TPI1*p, *PYK1p*, *PGI1*p and *TDH2*p) correspond to yeast glycolytic pathway genes and have been widely used to drive high gene expression when provided with glucose as a carbon source. An abundance of glucose in the media is catabolized by yeast mainly by fermentation, whereas cells assimilating glycerol respire as its catabolism provides electrons to the mitochondrial respiratory chain [23]. Our constitutive promoter library proved to drive expression across a high dynamic range with both carbon sources as assessed by anti-NifU WB of alkaline cell extracts. In particular, the promoters *PGK1*p, *TDH3*p and *TEF2*p drove very high expression of NifU in both glucose and glycerol. The promoters *TPI1*p, *PYK1p*, *PGI1*p, *TDH2*p and *HXT7*p, however, drove several-fold higher levels of expression of NifU with glycerol as the carbon source. The most dramatic difference was observed with *HXT7*p, which increased considerably in the case of cells that were respiring on glycerol, consistent with its role as a high-affinity hexose transporter and previously known to be induced at low glucose concentrations [24].

### Mitochondrial targeting signal library

**Fig. 3:**
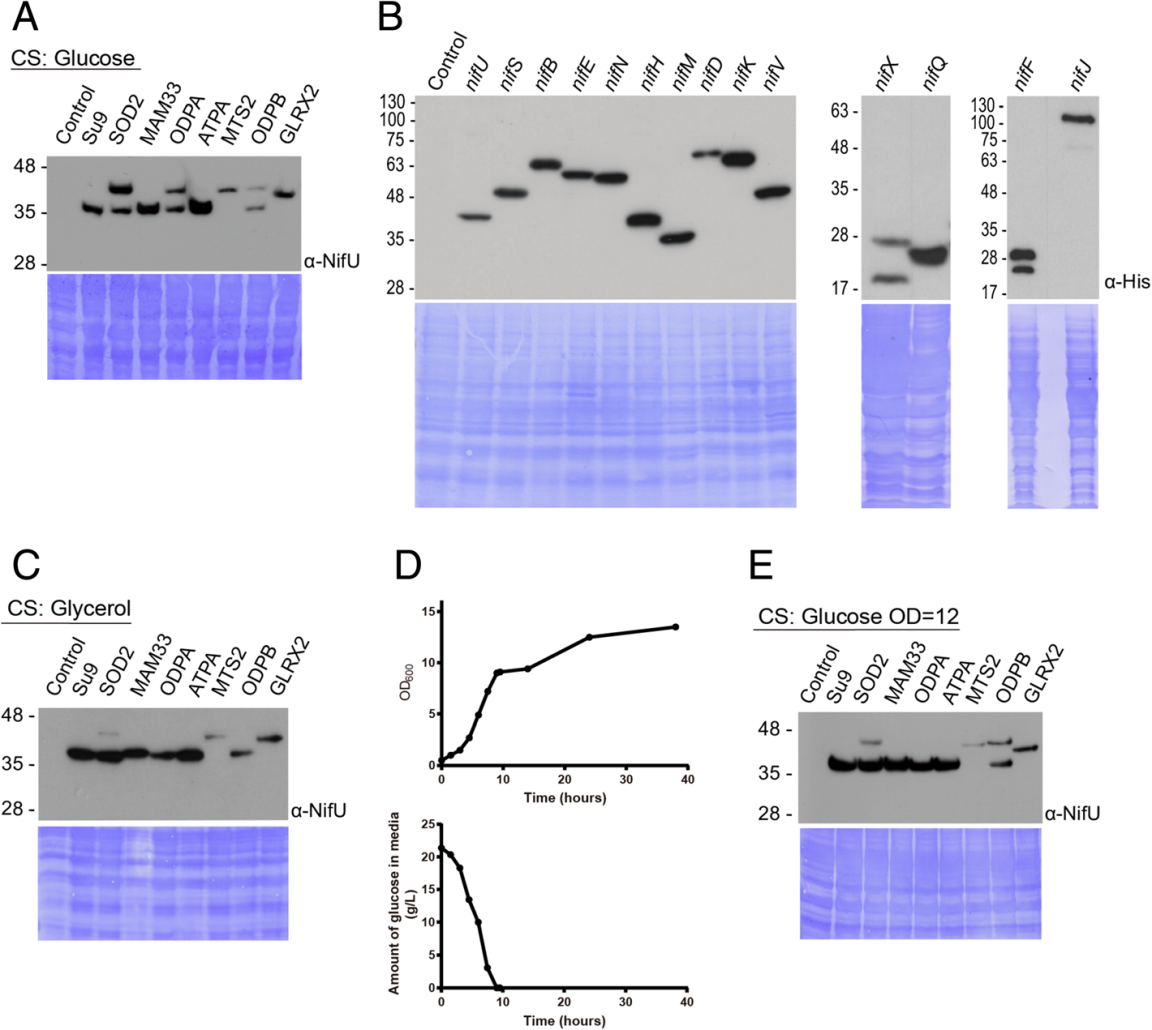
Processing of different mitochondrial targeting sequences fused to NifU in different culture conditions and analysis of *nif-*related gene expression. **a** Anti-NifU WB showing the processing of the different mitochondrial targeting signals of NifU (W303 strains with constructs inserted into *YPRCΔ15*) in exponentially growing cultures containing glucose as carbon source. The lower panel shows the corresponding membrane Coomassie stained as a loading control. **b** Anti-His WB analysis of *nif*-related gene expression in W303 exponentially growing cultures (two left panels) or saturated cultures (OD_600_ ~ 10, right panel) using glucose as the carbon source. All strains carried the *nif* genes inserted into the *YPRCΔ15* locus. The lower panels show the corresponding membrane Coomassie stained as a loading control. **c** Same as (**a**), but using glycerol as the carbon source. **d** Growth curve of a *S. cerevisiae* W303 culture growing with glucose as the carbon source (upper panel) and glucose determination in the media (lower panel). Note the growth lag that coincides with the exhaustion of glucose and with a probable adaptation of the culture to ethanol respiration. **e** Processing of different mitochondrial targeting signals of NifU in W303 cultures containing glucose as carbon source at an OD_600_ of 12 (post-glucose exhaustion). The lower panel shows the corresponding membrane Coomassie stained as a loading control

A library of MTSs was also included in the toolkit (Additional file 5: Figure S2) to avoid excessive repetition when expressing and targeting multiple genes to mitochondria. Apart from the two MTSs formerly demonstrated to target homologous proteins to the yeast mitochondrial matrix from the *N. crassa* Su9 protein [25] and from *S. cerevisiae* MAM33 [26] (Fig. 1c), we tested the mitochondrial import of NifU fused to a plant MTS (MTS2, from the β subunit of *Nicotiana plumbaginifolia* F1 ATPase, [27]) and five additional MTSs from *S. cerevisiae* proteins identified as part of the yeast mitochondrial proteome (GLRX2, ATPA, ODPA, ODPB and SOD2) [28]. Targeting of the tested MTSs into their proteolytically cleaved, higher mobility processed form was used as an indicator of import into the mitochondrial matrix as tested by anti-NifU WB. The sizes of the MTSs range from 8.5 to 22.5 kDa (for ODPA/ODPB and MTS2, respectively) and a higher mobility band detected by WB corresponding to a loss of the MTS is suggestive of import of the NifU protein into the mitochondrial matrix (Fig. 3a). For MTSs SOD2, ODPA and ODPB the cleavage was not complete and two bands were clearly visible, the lower mobility band corresponding to the size of the NifU protein plus the MTS used in each case. In the case of MTS2 and GLRX2 fused to NifU, we observed only a single lower mobility band corresponding to a failure in the cleavage of the targeting peptide and/or a failure of import into the mitochondrial matrix. The other three MTSs tested, Su9, MAM33 and ATPA, yielded a single higher mobility band suggestive of correct matrix import and cleavage. Mitochondrial extracts were prepared for these strains and targeting of NifU into the mitochondrial matrix compartment was confirmed (Additional file 6: Figure S4).

### Expression of a variety of Nif proteins

To demonstrate the ability of our yeast-adapted GoldenBraid system to assemble and successfully express a variety of TUs, we separately expressed 14 different His-tagged nitrogenase-related proteins and assessed their production by anti-His tag WB (Fig. 3b). A subset of structural proteins included: the MoFe protein (encoded by *nifD* and *nifK*), which is the catalytic component of nitrogenase containing the active-site FeMo-cofactor and the P-cluster, and the Fe protein (encoded by *nifH*), which acts as obligate electron donor to the MoFe protein. NifH is also required for P-cluster formation and FeMo-co biosynthesis [29]. Also included is NifM, a protein with similarity to prolyl isomerases, which is required for the maturation of NifH. A second subset of proteins required for the biosynthesis of the cofactors embedded in the structural proteins included: the Nif-specific [Fe–S] cluster biosynthetic proteins NifU and NifS; the FeMo-co biosynthetic proteins NifB [30], NifEN [31], and NifX [32], the homocitrate synthase NifV [33], and the molybdenum donor NifQ [34]. A third subset included the NifF flavodoxin that acts as electron donor to NifH and the NifJ pyruvate-flavodoxin oxidoreductase.

Yeast optimized codon usage versions of the 13 *Azotobacter vinelandii nif* genes and *Klebsiella oxytoca nifJ* were assembled into TUs (Additional file 5: Figure S2), inserted into the *YPRCΔ15* locus of *S. cerevisiae* and expressed under the same conditions shown in Fig. 1c for *nifU* (i.e. using the *TDH3* constitutive promoter and the Su9 MTS). Nif proteins NifU, NifS, NifB, NifE, NifN, NifH NifM, NifD, NifK, NifV, NifQ and NifJ were detected as single bands in anti-His tag WB analysis (Fig. 3b), indicating good expression, mitochondrial targeting and MTS cleavage. Importantly, the produced proteins were stable enough to accumulate in cells of aerobically-grown cultures despite these proteins being oxygen sensitive. Nif proteins NifX and NifF were detected as two bands by WB, suggestive of incomplete MTS processing. Expression levels varied slightly among the different Nif proteins as determined by WB band intensity, and NifF and NifJ were only detected in the stationary growth phase.

Mitochondrial morphology and activity in *S. cerevisiae* is closely linked to the metabolic activity of yeast [35]. We wondered if a respiratory state in which cells have many small and very active mitochondria would increase the efficiency of the MTSs. Figure 3c shows that this was indeed the case, since growth in media with glycerol as the carbon source generally improved MTS processing. The ODPA and ODPB MTSs were completely processed, and processing of SOD2 was very much enhanced. In fact, longer term cultures grown in media containing glucose, where it had been exhausted and respiration of ethanol is probably taking place (Fig. 3d), showed a similar behavior (Fig. 3e).

**Fig. 4:**
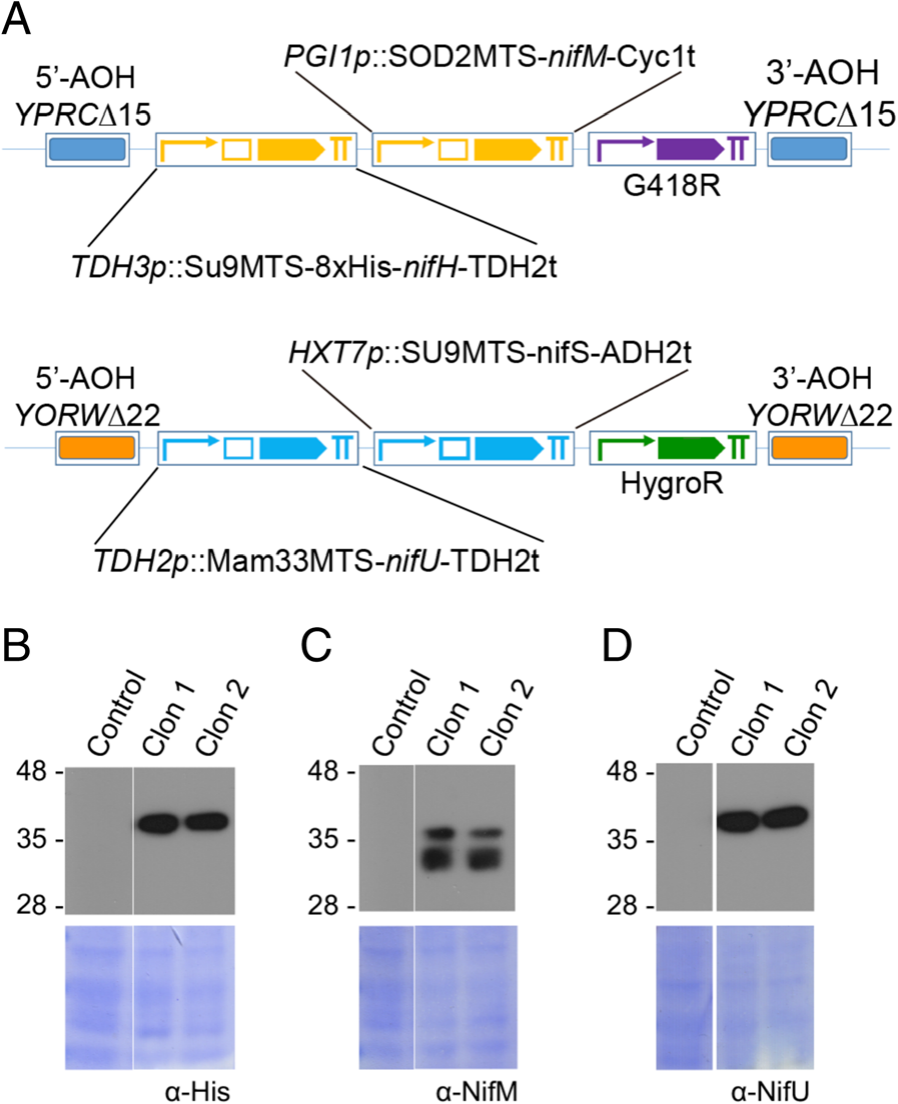
Example of the capabilities to generate genome-integrated multiple TU constructs using the GoldenBraid2.0 yeast-adapted system. **a** Diagram of the construct containing multiple TUs used for the simultaneous expression of *nifH*, *nifM, nifU* and *nifS*. WB analysis of NifH (**b**), NifM (**c**) and NifU (**d**) accumulation in exponentially growing cultures of the W303 strain containing glucose as the carbon source. The lower panel shows the corresponding membrane Coomassie stained as a loading control

To validate the potential of the GoldenBraid adapted system for the easy and rapid generation of *S. cerevisiae* strains with engineered pathways, we proceeded to generate more complex constructs containing three transcriptional units each and to generate a recombinant strain with insertions into both of the target sites. A construct containing the *nifU*, *nifS*, and the *hphMX* transcription units was generated and successfully inserted into the *YPRCΔ22* locus. Next, this strain was transformed with a construct containing transcriptional units for *nifH, nifM,* and *kanMX* for insertion into the *YORWΔ15* locus (Fig. 4a). Following the second integration in the genome, two doubly resistant colonies were tested and showed comparable levels of accumulation of the recombinant proteins (Fig. 4b-d) (note that NifS fails to accumulate since its expression is driven by the *HXT7*p that is not induced during growth in glucose, data not shown).

## Discussion

We describe the development and validation of a synthetic biology toolkit for modular assembly of TUs and their expression in yeast. This toolkit has a number of features that make it beneficial. We show the ability to assemble and genome integrate constructs containing multiple TUs with ease in a workflow that takes approximately 2 weeks (with an additional week if new GB parts are required). Our results are predominantly focused on the proof-of-principle expression of the [Fe–S] cluster biosynthetic protein NifU, which is fundamental to the synthesis of metallocluster cofactors of nitrogenase enzymes. We demonstrate consistent expression of *nifU* across individual transformants with no variation in expression as assessed by WB. Our system has recombination arms of homology for single copy integration at two solo LTR loci (*YPRCΔ15* and *YORWΔ22*) that we show support integration into two yeast strains, W303 and CEN.PK. It is expected that our system could also be used for integration in other strains of *S. cerevisiae* and even into other members of the *Saccharomyces* sensu stricto *clade*, given that they also contain these solo LTR loci (which can be highly divergent) and that the degree of homology at these loci are sufficient to support double recombination. Both integration target loci appear to yield similar levels of expression of *nifU* driven by the constitutive *TDH3* promoter. This could be advantageous as the expression level of a given integrated TU could solely be driven by the strength of its promoter, not by differences in the integration locus or copy number. Thus, in situations where the stoichiometry of proteins expressed from two separate TU assemblies is paramount, using the same promoter at both integrative sites would be expected to produce similar levels of protein. In contrast to this, promoters of different strengths are expected to have the same relative difference in strength regardless of insertion at either loci.

Our library of promoter parts for driving transgene expression give the option for induced expression via the *GAL1* promoter or constitutive expression using eight promoters that span a high dynamic range. A subset of these constitutive promoters showed different expression profiles during fermentative versus respiration growth. The promoters *TPI1*p, *PYK1p*, *PGI1*p, *TDH2*p and *HXT7*p resulted in higher expression in cultures with glycerol provided as the carbon source, facilitating respiration (Fig. 2b versus 2C). Most dramatically, the high affinity hexose transporter promoter, *HXT7*p, only drove detectable accumulation of NifU protein in glycerol-grown cells, a result that is likely related to this promoter being most active in hexose deprived conditions.

Taken together, our toolkit provides users with a wide flexibility of promoters to choose from that drive expression across a range of strengths and have differences in expression dependent on carbon source. This is especially significant in our case where we desire the import and accumulation of heterologous nitrogenase proteins specifically within the mitochondrial compartment and gives us additional promoter options to choose from in glycerol-grown respiring conditions. Of final note, it has been shown that yeast promoters are highly orthogonal since their relative strengths are largely independent of the downstream coding sequence [36]. The results observed for *nifU* are consistent with this, since the chosen promoters cover a range of expression similar to that shown in previous work with reporter genes [22]. The same expression behavior can thus be expected for other transgenes.

Our toolkit also contains a library of validated MTSs for targeting of heterologous proteins to the mitochondrial matrix. We observed an overall enhancement of mitochondrial import during respiring growth in the cultures, as assessed by a general increase in the cleaved, higher mobility protein bands on WBs. This suggests the favorable growth conditions for future attempts at expressing and assaying oxygen sensitive-recombinant protein function in yeast mitochondria. For two of the MTSs, SOD2 and MAM33, we confirmed that they function similarly for mitochondrial import independent of their cargo protein as the higher mobility MTS cleaved form of their cargo protein was consistently detected in cargo protein-specific WBs. For SOD2, compare the NifU doublet band pattern in the Fig. 3a WB versus the doublet seen in the NifM WB in Fig. 4b. Similarly for MAM33, compare the NifU single cleaved band pattern in the Fig. 3a WB versus the single band seen in the NifU WB in Fig. 4b. The consistency of import/cleavage conferred by any MTS upon a cargo protein should be confirmed for every protein fusion as it was established that the nature of the cargo protein can influence the efficiency of protein delivery and translocation across the mitochondrial membrane [37].

### Comparison with other modular assembly toolkits

We developed our yeast adapted GoldenBraid toolkit based on the GB2.0 standard. The current GoldenBraid standard is GB3.0 and incorporates new bioinformatics tools through the GoldenBraid website (www.gbcloning.org). Functionally, both of these GoldenBraid standards are fully compatible, with GB3.0 introducing a few new structural elements. GB3.0 includes a new set of destination vectors with increased *in planta* transformation efficiency and a CamR domestication vector, all of which work seamlessly with GB2.0 and our toolkit. In contrast to other cloning methods such as Gibson assembly, GoldenBraid requires the removal of specific Type IIS restriction sites from the parts in order for the GB reaction to proceed (termed “domestication”). Thus, the creation of a library of interchangeable and shareable parts is a key component of GB assembly, in contrast to Gibson assembly. Our toolkit has similar advantages to other modular assembly cloning toolkits used for yeast, such as MoClo-YTK and yGG [1, 2]. Unfortunately, the 4 nucleotide flanking overhangs that determine part type are different in all three systems, thus making parts incompatible between them. However, our choice of overhangs correspond with the agreed upon international standard for MoClo and GoldenBraid designs [38], making it possible to use any future part designed for these systems. In our system, the solo LTR-targeting arms of homology are fixed, allowing integration into two genomic loci. This is similar to the strategies employed by other toolkits, such as the MoClo-YTK toolkit that integrates constructs as a single copy into a landing pad at the *URA3* locus [1] or the yGG toolkit that allows for single copy integrations into the *URA3*, *LEU2*, *TRP1*, and/or *HIS3* loci [2]. Our choice to integrate into these two solo LTR loci was motivated by these sites being reported to support a high level of transgene expression, a finding verified by our results [14]. A design consideration that is not included in our system would be to include new recombination arms of homology as separate TU cassettes flanking the transgene-containing TUs. This design would be a way to allow the use of an assembly toolkit to build constructs that could be integrated into the genome of any organism that supports transformation and recombination of linear DNA fragments.

## Conclusions

We have validated the utility of the GoldenBraid modular assembly cloning system for heterologous gene expression in yeast by the integration of multiple TU-containing constructs into two genome loci. The assembly of such constructs in the yeast adapted system was rapid and produced consistent and repeatable results. Our goal was to create a system for mitochondrial targeting of heterologous proteins throughout a range of expression levels. Our evaluation of promoter and MTS parts demonstrated that several of each were effective in mediating a broad range of expression and for the import of recombinant proteins into the yeast mitochondrial matrix. By establishing the validity of this toolkit, further studies can now proceed towards the expression of active assemblies of nitrogenase enzymes in yeast mitochondria.

## Additional files

Additional file 1: Table S1. (.doc) List of primers and templates used for the domestication of parts. (DOCX 17 kb)

Additional file 2: Table S2. (.doc) Sequences of primers used (DOCX 15 kb)

Additional file 3: Figure S1. (.doc) Sequences of parts used for transcriptional unit assembly. (DOCX 25 kb)

Additional file 4: Figure S2. (.doc) Results of BLAST (yeastgenome.org/blast-sgd; matrix BLOSUM62) of the AOH sequences cloned in the adapted vectors and yeast strain W303 AND CEN-PK genomes. (DOCX 26 kb)

Additional file 5: Figure S3. (.doc) List of parts used for transcriptional unit assembly. (DOCX 179 kb)

Additional file 6: Figure S4. (.tiff) Mitochondrial fractionation from ATPA-NifU and MAM33-NifU cells. Lanes corresponding to total cell extract, pelleted cell debris after centrifugation, cytoplasmic and mitochondria-enriched fractions are shown. Anti-NifU WB is shown in the upper panel, anti-HSP60 WB (mitochondrial matrix marker) is the middle panel and anti-tubulin WB (cytoplasmic marker) is the lower panel. (PDF 677 kb)

Additional file 7: Figure S5. (.doc) Addgene ID numbers of the plasmids described in this work. (DOCX 13 kb)
