# Supplementary material for: Adaptation of the GoldenBraid modular cloning system and creation of a toolkit for the expression of heterologous proteins in yeast mitochondria
Source: BMC Biotechnol. 2017 Nov 13;17:80. doi: 10.1186/s12896-017-0393-y (PMC5683533; doi:10.1186/s12896-017-0393-y)
Supplement: Supplementary file 1 — (.doc) List of primers and templates used for the domestication of parts. (DOCX 17 kb) [file 12896_2017_393_MOESM1_ESM.docx]

**Adaptation of the GoldenBraid modular cloning system and creation of a toolkit for the expression of mitochondrial proteins in yeast.** Ana Pérez-González, Ryan Kniewel, Marcel Veldhuizen, Hemant K. Verma, Mónica Navarro-Rodríguez, Luis M. Rubio and Elena Caro.

**Table S1**

| **Part** | **Comment** | **Primers used for amplification** | **Template** |
| --- | --- | --- | --- |
| GAL1p | Promoter | 1516/1517/1518/1519 | pESC-Leu |
| TDH3p | Promoter | 1498/1499 | Yeast genome |
| TDH2p | Promoter | 1737/1738 | Yeast genome |
| TEF2p | Promoter | 1739/1740 | Yeast genome |
| HXT7p | Promoter | 1508/1509/1510/1511 | Yeast genome |
| TPI1p | Promoter | 1512/1513/1514/1515 | Yeast genome |
| PYK1p | Promoter | 1735/1736 | Yeast genome |
| PGK1p | Promoter | 1733/1734 | Yeast genome |
| PGI1p | Promoter | 1731/1732 | Yeast genome |
| Su9 MTS | mitochondrial targeting signal | 1492/1493 | Lopez-Torrejon et al (2016) |
| SOD2 MTS | mitochondrial targeting signal | 1490/1491 | Lopez-Torrejon et al (2016) |
| MAM33 MTS | mitochondrial targeting signal | 1494/1495 | Yeast genome |
| ODPA MTS | mitochondrial targeting signal | 2095/2096 | Yeast genome |
| ODPB MTS | mitochondrial targeting signal | 2097/2098 | Yeast genome |
| ATPA MTS | mitochondrial targeting signal | 2087/2088 | Yeast genome |
| GLRX2 MTS | mitochondrial targeting signal | 2093/2094 | Yeast genome |
| MTS2 | mitochondrial targeting signal | 1438/1439 | Synthesis by Genscript |
| *nifU* | Codon optimization for yeast of *Azotobacter vinelandii* gene | 1743/1744 | Synthesis by Genscript |
| *nifS* | Codon optimization for yeast of *Azotobacter vinelandii* gene | 1745/1746/1747/1748 | Synthesis by Genscript |
| *nifB* | Codon optimization for yeast of *Azotobacter vinelandii* gene | 1387/1389 | Synthesis by Genscript |
| *NifE* | Codon optimization for yeast of *Azotobacter vinelandii* gene | 2081/2082/2083/2984 | Synthesis by Genscript |
| *nifN* | Codon optimization for yeast of *Azotobacter vinelandii* gene | 2077/2078/2079/2080 | Synthesis by Genscript |
| *nifH* | Codon optimization for yeast of *Azotobacter vinelandii* gene | 1390/1392 | Synthesis by Proteogenix |
| *nifM* | Codon optimization for yeast of *Azotobacter vinelandii* gene | 1393/1395 | Synthesis by Genscript |
| *nifD* | Codon optimization for yeast of *Azotobacter vinelandii* gene | 1396/1398 | Synthesis by Genscript |
| *nifK* | Codon optimization for yeast of *Azotobacter vinelandii* gene | 1399/1400/1401/1403 | Synthesis by Genscript |
| *nifV* | Codon optimization for yeast of *Azotobacter vinelandii* gene | 2134/1641/1642/1643/1644/1645/1646/1647 | Synthesis by Genscript |
| *nifX* | Codon optimization for yeast of *Azotobacter vinelandii* gene | 2136/1651 | Synthesis by Genscript |
| *nifF* | Codon optimization for *Arabidopsis thaliana* of *Azotobacter vinelandii* gene | 2133/1633/1634/1635 | Synthesis by Genscript |
| *nifQ* | Codon optimization for yeast of *Azotobacter vinelandii* gene | 1862/1863/1864/1865 | Synthesis by Genscript |
| *nifJ* | Codon optimization for *Arabidopsis thaliana* of *Klebsiella pneumoniae* gene | 2158/2159/2160/2161 | Synthesis by Genscript |
| TDH2t | Terminator | 1522/1523 | Yeast genome |
| Cyc1t | Terminator | 1528/1529 | pESC-Leu |
| ADH2t | Terminator | 1530/1531/1532/1533 | Yeast genome |
| KanMX cassette | loxP-*Ag*TEFp-Sk*KanMX*-*Ag*TEFt-loxP | 1548/1550/1551/1549 | pUG6 |
| HygroR cassette | loxP-*Ag*TEFp-*Shhph*-*Ag*TEFt-loxP | 1548/1556/1557/1558/1559/1549 | pUG75 |
