# Supplementary material for: Adaptation of the GoldenBraid modular cloning system and creation of a toolkit for the expression of heterologous proteins in yeast mitochondria
Source: BMC Biotechnol. 2017 Nov 13;17:80. doi: 10.1186/s12896-017-0393-y (PMC5683533; doi:10.1186/s12896-017-0393-y)
Supplement: Supplementary file 2 — (.doc) Sequences of primers used (DOCX 15 kb) [file 12896_2017_393_MOESM2_ESM.docx]

**Adaptation of the GoldenBraid modular cloning system and creation of a toolkit for the expression of mitochondrial proteins in yeast.** Ana Pérez-González, Ryan Kniewel, Marcel Veldhuizen, Hemant K. Verma, Mónica Navarro-Rodríguez, Luis M. Rubio and Elena Caro.

**Table S2**

| **Primer** | **Sequence** |
| --- | --- |
| 1826 | AGCGGCCGCGAATTGGAGTTCGTCTTGTTATAA |
| 1827 | AGCGGCCGCCGCGTTGCTGGCGTTTTTC |
| 1828 | AGCGGCCGCCCAGCCAGGACAGAAATGCC |
| 1516 | GCGCCGTCTCGCTCGGGAGAGTACGGATTAGAAGCCGCC |
| 1517 | GCGCCGTCTCGACCGCTTCGCTGATTAATTAC |
| 1518 | GCGCCGTCTCGCGGTGATTTTTGATCTATTAACAG |
| 1519 | GCGCCGTCTCGCTCGATGGTGGGTTTTTTCTCCTTGACGT |
| 1498 | GCGCCGTCTCGCTCGGGAGCGAGTTTATCATTATCAATACTGC |
| 1499 | GCGCCGTCTCGCTCGATGGTTTGTTTGTTTATGTGTGTTTATTCG |
| 1737 | GCGCCGTCTCGCTCGGGAGATTGGTTTTTCCAGTGAATGATTATT |
| 1738 | GCGCCGTCTCGCTCGATGGTTTGTTTTGTTTGTTTGTGTGATGAA |
| 1739 | GCGCCGTCTCGCTCGGGAGGGGGCCGTATACTTACATATA |
| 1740 | GCGCCGTCTCGCTCGATGGGTTTAGTTAATTATAGTTCGTTGACC |
| 1508 | GCGCCGTCTCGCTCGGGAGCCGTGGAAATGAGGGGTATG |
| 1509 | GCGCCGTCTCGGCCTCTATTTATACTTTTTTTTTTTCAG |
| 1510 | GCGCCGTCTCGAGGCGATATATGCCAATACTTC |
| 1511 | GCGCCGTCTCGCTCGATGGTTTTTGATTAAAATTAAAAAAACTTTTTGTTTTTG |
| 1512 | GCGCCGTCTCGCTCGGGAGCTACGTATGGTCATTTCTTCTTC |
| 1513 | GCGCCGTCTCGATGTCTCCCTCACAATCAGTC |
| 1514 | GCGCCGTCTCGACATAACTACATAGTGTTTAAAGATTAC |
| 1515 | GCGCCGTCTCGCTCGATGGTTTTAGTTTATGTATGTGTTTTTTGTAGT |
| 1735 | GCGCCGTCTCGCTCGGGAGGAAAGTTTTTCCGGCAAGCTA |
| 1736 | GCGCCGTCTCGCTCGATGGTGTGATGATGTTTTATTTGTTTTGATTG |
| 1733 | GCGCCGTCTCGCTCGGGAGGAAGTACCTTCAAAGAATGGG |
| 1734 | GCGCCGTCTCGCTCGATGGTTGTTTTATATTTGTTGTAAAAAGTAGATAAT |
| 1731 | GCGCCGTCTCGCTCGGGAGGTAACAAAAATCACGATCTGGG |
| 1732 | GCGCCGTCTCGCTCGATGGTTTTAGGCTGGTATCTTGATTCTA |
| 1492 | GCGCCGTCTCGCTCGCCATGGCCTCCACTCGTGTCCT |
| 1493 | GCGCCGTCTCGCTCGCATTGAGGAAGAGTAGGCGCGCTTCT |
| 1534 | GCGCCGTCTCGCTCGCCATGTTCGCTAAGACCGCTGC |
| 1535 | GCGCCGTCTCGCTCGCATTGAAGATAACAATGATAAACCACCCTT |
| 1494 | GCGCCGTCTCGCTCGCCATGTTCTTAAGAAGCGTTAACCG |
| 1495 | GCGCCGTCTCGCTCGCATTGAGTTCCTCATGATTGCAGCGG |
| 2095 | GCGCCGTCTCGCTCGCCATGCTTGCTGCTTCATTCAAAC |
| 2096 | GCGCCGTCTCGCTCACATTGACATGGTACGAACATGACCTAT |
| 2097 | GCGCCGTCTCGCTCGCCATGTTTTCCAGACTGCCAACA |
| 2098 | GCGCCGTCTCGCTCACATTGAGAATCTCAATGCTGCTGCTG |
| 2087 | GCGCCGTCTCGCTCGCCATGTTGGCTCGTACTGCTGC |
| 2088 | GCGCCGTCTCGCTCACATTGACAATCTTCTGGTGGAAGCCA |
| 2093 | GCGCCGTCTCGCTCGCCATGGAGACAAATTTTTCCTTCGAC |
| 2094 | GCGCCGTCTCGCTCACATTGACATTTTTGGAGTAGATAAAAATCTTTTAG |
| 1438 | GCGCCGTCTCGCTCGAATGGCTTCTCGGAGGCTTCT |
| 1439 | GCGCCGTCTCGCTCGGGCTACCAGCGCCGGTGAACTCAT |
| 1743 | GCGCCGTCTCGCTCGAGCCATGTGGGACTACTCTGAAAAG |
| 1744 | GCGCCGTCTCGCTCGAAGCTCAGACTTCCATTTGGGCGT |
| 1745 | GCGCCGTCTCGCTCGAGCCATGGCCGACGTTTACTTGGA |
| 1746 | GCGCCGTCTCGCTATGTTAGCTGTGTTAGGTAATC |
| 1747 | GCGCCGTCTCGATAGCATTCGAATACATCGAAGG |
| 1748 | GCGCCGTCTCGCTCGAAGCTCAACCATAGACAGGAGCAAA |
| 1387 | GCGCCGTCTCGCTCGAGCCATGGAATTGTCTGTTTTGGGTC |
| 1389 | GCGCCGTCTCGCTCGAAGCTTAAGCCTTAGCTTGCAACAAAG |
| 2081 | GCGCCGTCTCGCTCGAGCCATGAAAGCTAAGGATATAGCGG |
| 2082 | GCGCCGTCTCGCTATGTTGTACTCCCCTATCA |
| 2083 | GCGCCGTCTCGATAGCTGGGGAATTTTGGCAT |
| 2084 | GCGCCGTCTCGCTCAAAGCTTAAGCATTAGCTGAACGAGC |
| 2077 | GCGCCGTCTCGCTCGAGCCATGGCTGAAATCATCAACAGAAAC |
| 2078 | GCGCCGTCTCGCTATGTATTCCAAGTCACCAG |
| 2079 | GCGCCGTCTCGATAGCTGAATCTATCGAATCATTC |
| 2080 | GCGCCGTCTCGCTCAAAGCTTAGTGTCTCCATTGTGGTTG |
| 1652 | GCGCCGTCTCGCTCGAGCCATGGCCATGAGACAATGTGC |
| 1653 | GCGCCGTCTCGCTCGAAGCTCATACTTCTTCAGCAGTTTTACC |
| 1390 | GCGCCGTCTCGCTCGAGCCATGGCCATGAGACAATGTGC |
| 1392 | GCGCCGTCTCGCTCGAAGCTCATACTTCTTCAGCAGTTTTACC |
| 1393 | GCGCCGTCTCGCTCGAGCCATGGCCTCAGAAAGATTAGCT |
| 1395 | GCGCCGTCTCGCTCGAAGCTTAACCATGTGCTAAGTTTTCCAA |
| 1396 | GCGCCGTCTCGCTCGAGCCATGACCGGTATGTCCCGCGAAGAAGTTGA |
| 1398 | GCGCCGTCTCGCTCGAAGCTCAGGCGGATGCGGCAACTT |
| 1399 | GCGCCGTCTCGCTCGAGCCATGTCTCAACAAGTTGATAAGATTAA |
| 1400 | GCGCCGTCTCGCATCTCTCTTTTTGGCTAACATAT |
| 1401 | GCGCCGTCTCGGATGGTTTCGAAGAAAAGTACC |
| 1403 | GCGCCGTCTCGCTCGAAGCTCATCTAACTAAGTCGTGGTTG |
| 2134 | GCGCCGTCTCGCTCGagccATGGCAAGTGTAATAATAGACGATACCACATTGAGAGATGGTGAACAA |
| 1641 | GCGCCGTCTCGCGTCGCACAACCTACACCAA |
| 1642 | GCGCCGTCTCGGACGTTGATCTCGCTGCAGC |
| 1643 | GCGCCGTCTCGCAAGACCTGCCATTCTAGCT |
| 1644 | GCGCCGTCTCGCTTGAGGTTTGTTTAGGATGC |
| 1645 | GCGCCGTCTCGGAAGACCATCCACGTGGATT |
| 1646 | GCGCCGTCTCGCTTCTCAAGCATAGAAGGAACTACGAGGGTCTTAACCCAGAT |
| 1647 | GCGCCGTCTCGCTCGAAGCTCAAGCCATACCACCAGCTG |
| 2136 | GCGCCGTCTCGCTCGagccATGAGTTCCCCAACAAGACAA |
| 1651 | GCGCCGTCTCGCTCGAAGCTCACTCATCCCAACCTTCTG |
| 2133 | GCGCCGTCTCGCTCGagccATGGCTAAGATTGGATTATTCTTC |
| 1633 | GCGCCGTCTCGCAAGACCAACGAACTTTCCAT |
| 1634 | GCGCCGTCTCGCTTGCACTCGATCTTGATAAC |
| 1635 | GCGCCGTCTCGCTCGAAGCTCAGAGACTCAAACCGAACTC |
| 1862 | GCGCCGTCTCGCTCGAGCCATGGGTTCCGCTGCCGCACA |
| 1863 | GCGCCGTCTCGCATCTCTTCTCATTTCCAACAAAT |
| 1864 | GCGCCGTCTCGGATGAATGGGAAGAATTGAGAG |
| 1865 | GCGCCGTCTCGCTCGAAGCTCAAATTTCTGCACCGAAACAATC |
| 2158 | GCGCCGTCTCGCTCGAGCCATGTCAGGAAAGATGAAAACTATGGAT |
| 2159 | GCGCCGTCTCGGTGTCTCTCCGCATCCAGCA |
| 2160 | GCGCCGTCTCGACACCATATGCTAGGTTAATCAC |
| 2161 | GCGCCGTCTCGCTCAAAGCTCACTCTTCTCCAGCAAGCC |
| 1522 | GCGCCGTCTCGCTCGGCTTTAAATTTAACTCCTTAAGTTACTTTAATG |
| 1523 | GCGCCGTCTCGCTCGAGCGGCGAAAAGCCAATTAGTGTGA |
| 1528 | GCGCCGTCTCGCTCGGCTTATCCGCTCTAACCGAAAAGG |
| 1529 | GCGCCGTCTCGCTCGAGCGCTTCGAGCGTCCCAAAACCT |
| 1548 | GCGCCGTCTCGCTCGGGAGCAGCTGAAGCTTCGTACGCT |
| 1549 | GCGCCGTCTCGCTCGAGCGGCATAGGCCACTAGTGGATC |
| 1550 | GCGCCGTCTCGGCGACGAAATACGCGATCGC |
| 1551 | GCGCCGTCTCGTCGCGCTCAGGCGCAATCAC |
| 1833 | AGGTGATTGAGTCTCTTGAAGTAC |
| 1836 | GGTTGTTTGGAGTCTATCTCTGAT |
| 1829 | TTTTTTCACGTCAGAAGTTAAGGC |
| 1832 | ATCTTGATTTGATGGGACTTCCTT |
| 1530 | GCGCCGTCTCGCTCGGCTTTAAGCGGATCTCTTATGTCTTTAC |
| 1531 | GCGCCGTCTCGATACGATTCAGAGGAGCAGG |
| 1532 | GCGCCGTCTCGGTATCCCTCGATATTTCTCATTTT |
| 1533 | GCGCCGTCTCGCTCGAGCGAATTATATAACTTGATGAGATGAGATG |
| 1556 | GCGCCGTCTCGGAAGACGCTGTCGAACTTTTC |
| 1557 | GCGCCGTCTCGCTTCGACCTGATGCAGCTCT |
| 1558 | GCGCCGTCTCGCGTCGCCTCGCTCCAGTCAA |
| 1559 | GCGCCGTCTCGGACGTTCGGGGATTCCCAAT |
