# Supplementary material for: Adaptation of the GoldenBraid modular cloning system and creation of a toolkit for the expression of heterologous proteins in yeast mitochondria
Source: BMC Biotechnol. 2017 Nov 13;17:80. doi: 10.1186/s12896-017-0393-y (PMC5683533; doi:10.1186/s12896-017-0393-y)
Supplement: Supplementary file 3 — (.doc) Sequences of parts used for transcriptional unit assembly. (DOCX 25 kb) [file 12896_2017_393_MOESM3_ESM.docx]

**Adaptation of the GoldenBraid modular cloning system and creation of a toolkit for the expression of mitochondrial proteins in yeast.** Ana Pérez-González, Ryan Kniewel, Marcel Veldhuizen, Hemant K. Verma, Mónica Navarro-Rodríguez, Luis M. Rubio and Elena Caro.

**Figure S1**

**>Gal1p:** promoter of *S. cerevisiae* YBR020W (455bp)

AGTACGGATTAGAAGCCGCCGAGCGGGTGACAGCCCTCCGAAGGAAGACTCTCCTCCGTGCGTCCTCGTCTTCACCGGTCGCGTTCCTGAAACGCAGATGTGCCTCGCGCCGCACTGCTCCGAACAATAAAGATTCTACAATACTAGCTTTTATGGTTATGAAGAGGAAAAATTGGCAGTAACCTGGCCCCACAAACCTTCAAATGAACGAATCAAATTAACAACCATAGGATGATAATGCGATTAGTTTTTTAGCCTTATTTCTGGGGTAATTAATCAGCGAAGCGATGATTTTTGATCTATTAACAGATATATAAATGCAAAAACTGCATAACCACTTTAACTAATACTTTCAACATTTTCGGTTTGTATTACTTCTTATTCAAATGTAATAAAAGTATCAACAAAAAATTGTTAATATACCTCTATACTTTAACGTCAAGGAGAAAAAACCCA

**>TDH3p:** promoter of *S. cerevisiae* YGR192C (675bp)

CGAGTTTATCATTATCAATACTGCCATTTCAAAGAATACGTAAATAATTAATAGTAGTGATTTTCCTAACTTTATTTAGTCAAAAAATTAGCCTTTTAATTCTGCTGTAACCCGTACATGCCCAAAATAGGGGGCGGGTTACACAGAATATATAACATCGTAGGTGTCTGGGTGAACAGTTTATTCCTGGCATCCACTAAATATAATGGAGCCCGCTTTTTAAGCTGGCATCCAGAAAAAAAAAGAATCCCAGCACCAAAATATTGTTTTCTTCACCAACCATCAGTTCATAGGTCCATTCTCTTAGCGCAACTACAGAGAACAGGGGCACAAACAGGCAAAAAACGGGCACAACCTCAATGGAGTGATGCAACCTGCCTGGAGTAAATGATGACACAAGGCAATTGACCCACGCATGTATCTATCTCATTTTCTTACACCTTCTATTACCTTCTGCTCTCTCTGATTTGGAAAAAGCTGAAAAAAAAGGTTGAAACCAGTTCCCTGAAATTATTCCCCTACTTGACTAATAAGTATATAAAGACGGTAGGTATTGATTGTAATTCTGTAAATCTATTTCTTAAACTTCTTAAATTCTACTTTTATAGTTAGTCTTTTTTTTAGTTTTAAAACACCAAGAACTTAGTTTCGAATAAACACACATAAACAAACAAA

**>TDH2p:** promoter of *S. cerevisiae* YJR009C (699bp)

TTGGTTTTTCCAGTGAATGATTATTTGTCGTTACCCTTTCGTAAAAGTTCAAACACGTTTTTAAGTATTGTTTAGTTGCTCTTTCGACATATATGATTATCCCTGCGCGGCTAAAGTTAAGGATGCAAAAAACATAAGACAACTGAAGTTAATTTACGTCAATTAAGTTTTCCAGGGTAATGATGTTTTGGGCTTCCACTAATTCAATAAGTATGTCATGAAATACGTTGTGAAGAGCATCCAGAAATAATGAAAAGAAACAACGAAACTGGGTCGGCCTGTTGTTTCTTTTCTTTACCACGTGATCTGCGGCATTTACAGGAAGTCGCGCGTTTTGCGCAGTTGTTGCAACGCAGCTACGGCTAACAAAGCCTAGTGGAACTCGACTGATGTGTTAGGGCCTAAAACTGGTGGTGACAGCTGAAGTGAACTATTCAATCCAATCATGTCATGGCTGTCACAAAGACCTTGCGGACCGCACGTACGAACACATACGTATGCTAATATGTGTTTTGATAGTACCCAGTGATCGCAGACCTGCAATTTTTTTGTAGGTTTGGAAGAATATATAAAGGTTGCACTCATTCAAGATAGTTTTTTTCTTGTGTGTCTATTCATTTTATTATTGTTTGTTTAAATGTTAAAAAAACCAAGAACTTAGTTTCAAATTAAATTCATCACACAAACAAACAAAACAAA

**>TEF2p:** promoter of *S. cerevisiae* YBR118W (520bp)

GCTTCCCCTGCCGGCTGTGAGGGCGCCATAACCAAGGTATCTATAGACCGCCAATCAGCAAACTACCTCCGTACATTCATGTTGCACCCACACATTTATACACCCAGACCGCGACAAATTACCCATAAGGTTGTTTGTGACGGCGTCGTACAAGAGAACGTGGGAACTTTTTAGGCTCACCAAAAAAGAAAGAAAAAATACGAGTTGCTGACAGAAGCCTCAAGAAAAAAAAAATTCTTCTTCGACTATGCTGGAGGCAGAGATGATCGAGCCGGTAGTTAACTATATATAGCTAAATTGGTTCCATCACCTTCTTTTCTGGTGTCGCTCCTTCTAGTGCTATTTCTGGCTTTTCCTATTTTTTTTTTTCCATTTTTCTTTCTCTCTTTCTAATATATAAATTCTCTTGCATTTTCTATTTTTCTCTCTATCTATTCTACTTGTTTATTCCCTTCAAGGTTTTTTTTTAAGGAGTACTTGTTTTTAGAATATACGGTCAACGAACTATAATTAACTAAAC

**>HXT7p:** promoter of *S. cerevisiae* YDR342C (587bp)

CCGTGGAAATGAGGGGTATGCAGGAATTTGTGCGGGGTAGGAAATCTTTTTTTTTTTTAGGAGGAACAACTGGTGGAAGAATGCCCACACTTCTCAGAAATGCATGCAGTGGCAGCACGCTAATTCGAAAAAATTCTCCAGAAAGGCAACGCAAAATTTTTTTTCCAGGGAATAAACTTTTTATGACCCACTACTTCTCGTAGGAACAATTTCGGGCCCCTGCGTGTTCTTCTGAGGTTCATCTTTTACATTTGCTTCTGCTGGATAATTTTCAGAGGCAACAAGGAAAAATTAGATGGCAAAAAGTCGTCTTTCAAGGAAAAATCCCCACCATCTTTCGAGATCCCCTGTAACTTATTGGCAACTGAAAGAATGAAAAGGAGGAAAATACAAAATATACTAGAACTGAAAAAAAAAAAGTATAAATAGAGACGATATATGCCAATACTTCACAATGTTCGAATCTATTCTTCATTTGCAGCTATTGTAAAATAATAAAACATCAAGAACAAACAAGCTCAACTTGTCTTTTCTAAGAACAAAGAATAAACACAAAAACAAAAAGTTTTTTTAATTTTAATCAAAAA

**>TPI1p:** promoter of *S. cerevisiae* YDR050C (918bp)

CTACGTATGGTCATTTCTTCTTCAGATTCCCTCATGGAGAAAGTGCGGCAGATGTATATGACAGAGTCGCCAGTTTCCAAGAGACTTTATTCAGGCACTTCCATGATAGGCAAGAGAGAAGACCCAGAGATGTTGTTGTCCTAGTTACACATGGTATTTATTCCAGAGTATTCCTGATGAAATGGTTTAGATGGACATACGAAGAGTTTGAATCGTTTACCAATGTTCCTAACGGGAGCGTAATGGTGATGGAACTGGACGAATCCATCAATAGATACGTCCTGAGGACCGTGCTACCCAAATGGACTGATTGTGAGGGAGACCTAACTACATAGTGTTTAAAGATTACGGATATTTAACTTACTTAGAATAATGCCATTTTTTTGAGTTATAATAATCCTACGTTAGTGTGAGCGGGATTTAAACTGTGAGGACCTTAATACATTCAGACACTTCTGCGGTATCACCCTACTTATTCCCTTCGAGATTATATCTAGGAACCCATCAGGTTGGTGGAAGATTACCCGTTCTAAGACTTTTCAGCTTCCTCTATTGATGTTACACCTGGACACCCCTTTTCTGGCATCCAGTTTTTAATCTTCAGTGGCATGTGAGATTCTCCGAAATTAATTAAAGCAATCACACAATTCTCTCGGATACCACCTCGGTTGAAACTGACAGGTGGTTTGTTACGCATGCTAATGCAAAGGAGCCTATATACCTTTGGCTCGGCTGCTGTAACAGGGAATATAAAGGGCAGCATAATTTAGGAGTTTAGTGAACTTGCAACATTTACTATTTTCCCTTCTTACGTAAATATTTTTCTTTTTAATTCTAAATCAATCTTTTTCAATTTTTTGTTTGTATTCTTTTCTTGCTTAAATCTATAACTACAAAAAACACATACATAAACTAAAA

**>PYK1p:** promoter of *S. cerevisiae* YAL038W (727bp)

GAAAGTTTTTCCGGCAAGCTAAATGGAAAAAGGAAAGATTATTGAAAGAGAAAGAAAGAAAAAAAAAAAATGTACACCCAGACATCGGGCTTCCACAATTTCGGCTCTATTGTTTTCCATCTCTCGCAACGGCGGGATTCCTCTATGGCGTGTGATGTCTGTATCTGTTACTTAATCCAGAAACTGGCACTTGACCCAACTCTGCCACGTGGGTCGTTTTGCCATCGACAGATTGGGAGATTTTCATAGTAGAATTCAGCATGATAGCTACGTAAATGTGTTCCGCACCGTCACAAAGTGTTTTCTACTGTTCTTTCTTCTTTCGTTCATTCAGTTGAGTTGAGTGAGTGCTTTGTTCAATGGATCTTAGCTAAAATGCATATTTTTTCTCTTGGTAAATGAATGCTTGTGATGTCTTCCAAGTGATTTCCTTTCCTTCCCATATGATGCTAGGTACCTTTAGTGTCTTCCTAAAAAAAAAAAAAGGCTCGCCATCAAAACGATATTCGTTGGCTTTTTTTTCTGAATTATAAATACTCTTTGGTAACTTTTCATTTCCAAGAACCTCTTTTTTCCAGTTATATCATGGTCCCCTTTCAAAGTTATTCTCTACTCTTTTTCATATTCATTCTTTTTCATCCTTTGGTTTTTTATTCTTAACTTGTTTATTATTCTCTCTTGTTTCTATTTACAAGACACCAATCAAAACAAATAAAACATCATCACA

**>PGK1p**: promoter of *S. cerevisiae* YCR012W (750bp)

ACGCACAGATATTATAACATCTGCATAATAGGCATTTGCAAGAATTACTCGTGAGTAAGGAAAGAGTGAGGAACTATCGCATACCTGCATTTAAAGATGCCGATTTGGGCGCGAATCCTTTATTTTGGCTTCACCCTCATACTATTATCAGGGCCAGAAAAAGGAAGTGTTTCCCTCCTTCTTGAATTGATGTTACCCTCATAAAGCACGTGGCCTCTTATCGAGAAAGAAATTACCGTCGCTCGTGATTTGTTTGCAAAAAGAACAAAACTGAAAAAACCCAGACACGCTCGACTTCCTGTCTTCCTATTGATTGCAGCTTCCAATTTCGTCACACAACAAGGTCCTAGCGACGGCTCACAGGTTTTGTAACAAGCAATCGAAGGTTCTGGAATGGCGGGAAAGGGTTTAGTACCACATGCTATGATGCCCACTGTGATCTCCAGAGCAAAGTTCGTTCGATCGTACTGTTACTCTCTCTCTTTCAAACAGAATTGTCCGAATCGTGTGACAACAACAGCCTGTTCTCACACACTCTTTTCTTCTAACCAAGGGGGTGGTTTAGTTTAGTAGAACCTCGTGAAACTTACATTTACATATATATAAACTTGCATAAATTGGTCAATGCAAGAAATACATATTTGGTCTTTTCTAATTCGTAGTTTTTCAAGTTCTTAGATGCTTTCTTTTTCTCTTTTTTACAGATCATCAAGGAAGTAATTATCTACTTTTTACAACAAATATAAAACA

**>PGI1p:** promoter of *S. cerevisiae* YBR196C (402bp)

GGTAACAAAAATCACGATCTGGGTGGGTGTGGGTGTATTGGATTATAGGAAGCCACGCGCTCAACCTGGAATTACAGGAAGCTGGTAATTTTTTGGGTTTGCAATCATCACCATCTGCACGTTGTTATAATGTCCCGTGTCTATATATATCCATTGACGGTATTCTATTTTTTTGCTATTGAAATGAGCGTTTTTTGTTACTACAATTGGTTTTACAGACGGAATTTTCCCTATTTGTTTCGTCCCATTTTTCCTTTTCTCATTGTTCTCATATCTTAAAAAGGTCCTTTCTTCATAATCAATGCTTTCTTTTACTTAATATTTTACTTGCATTCAGTGAATTTTAATACATATTCCTCTAGTCTTGCAAAATCGATTTAGAATCAAGATACCAGCCTAAAA

**>SU9 MTS:** from *Neurospora crassa* ATP synthase subunit 9 (OR74A)

ATGGCCTCCACTCGTGTCCTCGCCTCTCGCCTGGCCTCCCAGATGGCTGCTTCCGCCAAGGTTGCCCGCCCTGCTGTCCGCGTTGCTCAGGTCAGCAAGCGCACCATCCAGACTGGCTCCCCCCTCCAGACCCTCAAGCGCACCCAGATGACCTCCATCGTCAACGCCACCACCCGCCAGGCTTTCCAGAAGCGCGCCTACTCTTCC

**>SOD2 MTS:** from *S. cerevisiae* Superoxide dismutase [Mn] (YHR008C)

ATGTTCGCTAAGACCGCTGCTGCTAACTTAACTAAAAAGGGTGGTTTGTCATTGTTGAGTATGTTCGCCAAGACCGCCGCCGCAAATTTGACCAAAAAGGGTGGTTTATCATTGTTATCT

**>MAM33 MTS:** from *S. cerevisiae* Mitochondrial acidic protein (YIL070C)

ATGTTCTTAAGAAGCGTTAACCGTGCCGTCACTAGAAGCATTTTGACTACGCCCAAGCCAGCCGTAGTAAAATCATCGTGGAGAGTTTTTACTGTTGCTAACTCTAAGAGATGTTTCACACCCGCTGCAATCATGAGGAAC

**>ODPA MTS:** from *S. cerevisiae* Pyruvate dehydrogenase E1 component subunit alpha (YER178W)

ATGCTTGCTGCTTCATTCAAACGCCAACCATCACAATTGGTCCGCGGGTTAGGAGCTGTTCTTCGCACTCCCACCAGGATAGGTCATGTTCGTACCATG

**>ODPB MTS:** from *S. cerevisiae* Pyruvate dehydrogenase E1 component subunit beta (YBR221C)

ATGTTTTCCAGACTGCCAACATCATTGGCCAGAAATGTTGCACGTCGTGCCCCAACTTCTTTTGTAAGACCCTCTGCAGCAGCAGCAGCATTGAGATTC

**>ATPA MTS:** from *S. cerevisiae* ATP synthase subunit alpha (YBL099W)

ATGTTGGCTCGTACTGCTGCTATTCGTTCTCTATCGAGAACTCTAATTAACTCTACCAAGGCCGCAAGACCTGCCGCTGCTGCTTTGGCTTCCACCAGAAGATTG

**>GLRX2 MTS:** from *S. cerevisiae* Glutaredoxin-2 (YDR513W)

ATGGAGACCAATTTTTCCTTCGACTCGAATTTAATTGTTATTATCATTATCACGTTGTTTGCCACAAGAATTATTGCTAAAAGATTTTTATCTACTCCAAAAATG

**>MTS2 MTS:** from β subunit of *Nicotiana plumbaginifolia* F1 ATPase

ATGGCTTCTCGGAGGCTTCTCGCCTCTCTCCTCCGTCAATCGGCTCAACGTGGCGGCGGTCTAATTTCCCGATCGTTAGGAAACTCCATCCCTAAATCCGCTTCACGCGCCTCTTCACGCGCATCCCCTAAGGGATTCCTCTTAAACCGCGCCGTACAGTACGCTACCTCCGCAGCGGCACCGGCATCTCAGCCATCAACACCACCAAAGTCCGGCAGTGAACCGTCCGGAAAAATTACCGATGAGTTCACCGGCGCTGGT

**>yeast codon optimized *Azotobacter vinelandii* nifU (Genscript)**

ATGTGGGACTACTCTGAAAAGGTTAAGGAACATTTCTACAATCCAAAGAACGCCGGTGCTGTAGAAGGTGCAAACGCCATTGGTGACGTTGGTTCATTATCCTGTGGTGACGCTTTGAGATTAACATTGAAAGTTGACCCTGAAACCGATGTCATCTTGGACGCAGGTTTTCAAACTTTCGGTTGCGGTTCTGCTATTGCATCTTCATCCGCTTTGACTGAAATGGTTAAGGGTTTGACATTGGATGAAGCATTGAAAATCTCAAACCAAGATATCGCTGACTATTTGGATGGTTTGCCACCTGAAAAGATGCATTGTTCCGTCATGGGTAGAGAAGCCTTACAAGCTGCAGTAGCTAACTACAGAGGTGAAACCATTGAAGATGACCACGAAGAAGGTGCATTGATATGTAAATGCTTTGCCGTTGATGAAGTTATGGTCAGAGATACCATAAGAGCAAATAAGTTAAGTACTGTAGAAGATGTTACTAACTACACAAAAGCTGGTGGTGGTTGTTCTGCTTGCCATGAAGCAATAGAAAGAGTTTTGACAGAAGAATTGGCCGCTAGAGGTGAAGTATTCGTTGCAGCCCCAATTAAAGCCAAAAAGAAAGTCAAGGTATTGGCTCCAGAACCTGCCCCAGCTCCTGTTGCAGAAGCCCCAGCTGCAGCCCCTAAGTTGTCAAATTTGCAAAGAATTAGAAGAATCGAAACAGTCTTGGCTGCAATAAGACCTACCTTGCAAAGAGACAAAGGTGACGTCGAATTAATTGATGTAGACGGTAAAAATGTTTACGTCAAATTGACCGGTGCTTGTACTGGTTGCCAAATGGCATCCATGACATTAGGTGGTATACAACAAAGATTGATCGAAGAATTGGGTGAGTTCGTCAAAGTTATCCCAGTCTCCGCTGCCGCACACGCCCAAATGGAAGTCTGA

**>yeast codon optimized *Azotobacter vinelandii* nifS (Genscript)**

ATGGCCGACGTTTACTTGGATAATAACGCTACTACAAGAGTCGATGACGAAATAGTACAAGCTATGTTGCCATTTTTCACAGAACAATTCGGTAACCCTTCCAGTTTGCATTCCTTCGGTAACCAAGTTGGTATGGCCTTGAAGAAAGCTAGACAATCTGTCCAAAAATTGTTAGGTGCAGAACACGATTCCGAAATCGTTTTTACCAGTTGTGGTACTGAATCTGACTCAACCGCCATTTTGTCTGCCTTAAAAGCTCAACCAGAAAGAAAGACTGTCATAACCACTGTTGTCGAACATCCTGCAGTATTGTCTTTATGCGATTATTTGGCCTCAGAAGGTTACACTGTTCATAAGTTACCAGTCGATAAAAAGGGTAGATTGGACTTAGAACACTATGCTTCCTTGTTAACAGATGACGTAGCTGTAGTTAGTGTTATGTGGGCAAATAACGAAACTGGTACATTGTTTCCAATTGAAGAAATGGCAAGATTAGCCGATGACGCTGGTATAATGTTCCATACTGATGCAGTACAAGCCGTTGGTAAAGTCCCTATAGACTTGAAGAACTCGTCAATCCACATGTTGTCCTTAAGTGGTCATAAATTGCACGCTCCAAAGGGTGTTGGTGTCTTGTACTTAAGAAGAGGTACAAGATTCAGACCTTTGTTAAGAGGTGGTCATCAAGAAAGAGGTAGAAGAGCCGGTACTGAAAATGCTGCATCTATTATAGGTTTGGGTGTTGCCGCTGAAAGAGCTTTACAATTCATGGAACATGAAAACACTGAAGTTAAGAGATTGCGTGATAAGTTAGAAGCAGGTATTTTGGCCGTCGTACCACACGCATTTGTTACTGGTGACCCAGACAATAGATTACCTAACACAGCTAACATCGCATTCGAATACATCGAAGGTGAAGCTATCTTGTTGTTGTTGAACAAAGTTGGTATAGCAGCCTCCAGTGGTTCTGCTTGTACATCTGGTTCATTGGAACCATCACATGTTATGAGAGCAATGGATATTCCTTATACAGCTGCACACGGTACTGTTAGATTTTCTTTGAGTAGATACACAACCGAAGAAGAAATTGATAGAGTCATTAGAGAAGTACCACCTATTGTTGCTCAATTGAGAAAATTGTCTCCTTACTGGTCAGGTAATGGTCCTGTTGAAGACCCTGGTAAAGCCTTTGCTCCTGTCTATGGTTGA

**>yeast codon optimized *Azotobacter vinelandii* nifB (Genscript)**

ATGGAATTGTCTGTTTTGGGTCAAAACAACGGTGGTCAACACTCTGCTGGTGGTTGTTCTTCTTCTTCTTGTGGTTCTACTCACGACCAATTGTCTCACTTGCCAGAAAACATCAGAGCTAAGGTTCAAAACCACCCATGTTACTCTGAAGAAGCTCACCACTACTTCGCTAGAATGCACGTTGCTGTTGCTCCAGCTTGTAACATCCAATGTCACTACTGTAACAGAAAGTACGACTGTGCTAACGAATCTAGACCAGGTGTTGTTTCTGAAGTTTTGACTCCAGAACAAGCTGTTAAGAAGGTTAAGGCTGTTGCTGCTGCTATCCCACAAATGTCTGTTTTGGGTATCGCTGGTCCAGGTGACCCATTGGCTAACCCAAAGAGAACTTTGGACACTTTCAGAATGTTGTCTGAACAAGCTCCAGACATCAAGTTGTGTGTTTCTACTAACGGTTTGGCTTTGCCAGAATGTGTTGAAGAATTGGCTAAGCACAACATCGACCACGTTACTATCACTATCAACTGTGTTGACCCAGAAATCGGTGCTAAGATCTACCCATGGATCTACTGGAACAACAAGAGAATCAGAGGTGTTAAGGCTGCTAAGATCTTGATCGAACAACAACAAAAGGGTTTGGAAATGTTGGTTGCTAGAGGTATCTTGGTTAAGGTTAACTCTGTTATGATCCCAGGTGTTAACGACGAACACTTGAAGGAAGTTTCTAAGATCGTTAAGGCTAAGGGTGCTTTCTTGCACAACGTTATGCCATTGATCGCTGAACCAGAACACGGTACTTTCTACGGTGTTATGGGTCAAAGATCTCCAGAACCAGAAGAATTGCAAGACTTGCAAGACGCTTGTGCTGGTGACATGAACATGATGAGACACTGTAGACAATGTAGAGCTGACGCTGTTGGTATGTTGGGTGAAGACAGAGGTGACGAATTCACTTTGGACAAGATCGAATCTATGGAAATCGACTACGAAGCTGCTATGGTTAAGAGAGCTGCTATCCACGCTGCTATCAAGGAAGAATTGGACGAAAAGGCTGCTAAGAAGGAAAGATTGGCTGGTTTGTCTGTTGCTTCTGTTCAAAACGGTACTTCTGGTAGATACAGACCAGTTTTGATGGCTGTTGCTACTTCTGGTGGTGGTTTGATCAACCAACACTTCGGTCACGCTACTGAATTCTTGGTTTACGAAGCTTCTCCATCTGGTGTTAGATTCATCGGTCACAGAAGAGTTGACCAATACTGTGTTGGTAACGACACTTGTGGTGAAAAGGAATCTGCTTTGGCTGGTTCTATCAGAGCTTTGAAGGGTTGTGAAGCTGTTTTGTGTTCTAAGATCGGTTTCGAACCATGGTCTGACTTGGAAACTGCTGGTATCCAACCAAACGGTGAACACGCTATGGAACCAATCGAAGAAGCTGTTATGGCTGTTTACAGAGAAATGATCGAATCTGGTAGATTGGAAAACGACGGTGCTTTGTTGCAAGCTAAGGCTTAA

**>yeast codon optimized *Azotobacter vinelandii* nifE (MIT)**

ATGAAAGCTAAGGATATAGCGGAGTTATTAGATGAACCCGCTTGTTCACACAACAAGAAAGAAAAGTCAGGATGTGCTAAACCAAAGCCAGGGGCTACCGACGGTGGGTGTTCCTTCGATGGTGCTCAAATCGCTTTGCTTCCCGTCGCTGATGTTGCTCATATAGTACATGGACCGATTGCCTGCGCGGGTTCAAGTTGGGACAATAGAGGCACCAGGAGTTCAGGCCCGGATTTGTATAGGATTGGGATGACTACAGACTTAACTGAAAACGATGTTATAATGGGTAGAGCAGAAAAAAGATTGTTCCATGCCATCCGTCAAGCGGTTGAATCTTACTCTCCTCCAGCCGTCTTTGTATACAATACCTGTGTTCCAGCATTGATTGGCGACGATGTAGATGCCGTCTGCAAAGCAGCAGCAGAACGTTTTGGCACGCCGGTCATACCCGTCGATTCAGCAGGCTTTTATGGTACAAAGAATCTTGGCAACAGAATTGCAGGCGAAGCAATGTTAAAATACGTTATTGGTACGAGAGAACCAGACCCACTTCCAGTTGGCAGCGAACGTCCCGGGATCAGAGTACATGATGTTAATTTGATAGGGGAGTACAACATCGCTGGGGAATTTTGGCATGTTCTGCCTTTGCTAGATGAATTGGGATTGAGAGTCTTATGTACGTTGGCTGGTGACGCGAGGTACAGAGAAGTTCAGACGATGCATAGAGCCGAAGTGAACATGATGGTATGTTCTAAGGCTATGTTGAATGTTGCGAGAAAATTGCAAGAGACTTACGGAACCCCCTGGTTTGAAGGAAGCTTTTATGGTATAACCGATACGAGTCAGGCTTTGCGTGATTTTGCTAGACTATTAGACGATCCAGACCTGACTGCGCGTACGGAAGCGTTAATCGCTAGGGAAGAAGCTAAAGTACGTGCTGCATTGGAACCATGGAGGGCTAGGCTAGAGGGTAAAAGAGTATTGTTGTACACCGGCGGTGTCAAATCATGGTCTGTTGTTAGCGCCCTGCAAGACCTAGGTATGAAAGTCGTAGCGACAGGCACGAAAAAGTCTACAGAAGAGGACAAAGCAAGAATAAGAGAATTGATGGGTGATGACGTAAAGATGCTGGACGAGGGCAACGCCCGTGTATTGTTGAAAACAGTAGATGAATACCAAGCGGATATCTTAATAGCAGGTGGCAGGAACATGTATACGGCATTGAAGGGTCGTGTTCCTTTTTTAGACATTAACCAAGAAAGAGAATTTGGCTATGCAGGTTACGATGGTATGTTGGAACTAGTTCGTCAGCTTTGTATAACACTAGAATGTCCTGTGTGGGAGGCAGTCAGAAGGCCAGCACCCTGGGACATTCCTGCTAGTCAAGATGCCGCCCCATCAGCACCGGCTCGTTCAGCTAATGCTTAA

**>yeast codon optimized *Azotobacter vinelandii* nifN (MIT)**

ATGGCTGAAATCATCAACAGAAACAAAGCATTAGCAGTATCTCCATTAAAAGCATCACAAACTATGGGTGCCGCATTAGCAATCTTGGGTTTAGCAAGATCTATGCCATTGTTCCATGGTTCACAAGGTTGTACAGCCTTTGCTAAAGTTTTCTTTGTAAGACACTTCAGAGAACCAGTTCCTTTACAAACTACAGCTATGGATCAAGTCTCTTCAGTTATGGGTGCTGACGAAAATGTTGTAGAAGCATTAAAGACTATCTGTGAAAGACAAAACCCATCTGTTATTGGTTTGTTAACCACTGGTTTATCAGAAACTCAAGGTTGCGATTTGCATACAGCTTTGCACGAATTCAGAACCCAATACGAAGAATACAAGGATGTTCCAATCGTTCCTGTAAATACACCTGACTTTTCTGGTTGTTTCGAATCAGGTTTTGCTGCAGCCGTAAAAGCTATCGTCGAAACCTTGGTTCCAGAAAGAAGAGATCAAGTTGGTAAAAGACCTAGACAAGTCAATGTTTTGTGCTCTGCAAACTTAACACCTGGTGACTTGGAATACATCGCTGAATCTATCGAATCATTCGGTTTGCGACCATTGTTAATTCCTGACTTATCCGGTAGTTTGGATGGTCATTTGGACGAAAATAGATTCAACGCATTAACAACCGGTGGTTTGTCAGTTGCCGAATTAGCAACTGCCGGTCAATCCGTAGCAACATTGGTCGTTGGTCAAAGTTTAGCTGGTGCTGCAGATGCTTTGGCAGAAAGAACTGGTGTACCAGACAGAAGATTTGGCATGTTATATGGTTTGGATGCAGTTGACGCCTGGTTAATGGCCTTGGCTGAAATTTCCGGTAACCCAGTTCCTGATAGATACAAGAGACAAAGAGCACAATTGCAAGATGCCATGTTAGACACCCATTTTATGTTATCCAGTGCTAGAACTGCAATAGCCGCTGATCCAGACTTGTTATTGGGTTTCGATGCCTTATTGAGATCTATGGGTGCTCATACAGTCGCAGCCGTAGTCCCTGCTAGAGCTGCAGCCTTGGTTGATTCCCCATTACCTAGTGTAAGAGTCGGTGACTTGGAGGACTTGGAACATGCTGCAAGAGCCGGTCAAGCTCAATTGGTTATCGGTAATTCCCACGCATTGGCCAGTGCTAGAAGATTAGGTGTTCCATTATTGAGAGCAGGTTTTCCTCAATATGATTTGTTGGGTGGTTTCCAAAGATGCTGGTCTGGTTACAGAGGTTCTTCACAAGTATTGTTCGATTTGGCTAACTTATTGGTCGAACATCACCAAGGTATACAACCTTATCACTCAATCTACGCTCAAAAACCTGCTACAGAACAACCACAATGGAGACACTAA

**>yeast codon optimized *Azotobacter vinelandii* nifH (Proteogenix)**

ATGGCTATGAGACAATGTGCAATCTATGGTAAAGGTGGTATCGGTAAATCTACTACAACCCAAAATTTGGTTGCTGCATTAGCTGAAATGGGTAAAAAGGTTATGATTGTCGGTTGCGATCCAAAAGCTGACTCTACTAGATTGATCTTACACTCAAAGGCACAAAACACTATTATGGAAATGGCCGCTGAAGCCGGTACAGTAGAAGATTTGGAATTAGAAGACGTTTTGAAAGCAGGTTACGGTGGTGTAAAGTGTGTTGAATCAGGTGGTCCAGAACCTGGTGTTGGTTGCGCCGGTAGAGGTGTCATAACAGCTATTAATTTCTTGGAAGAAGAAGGTGCTTACGAAGATGACTTAGATTTCGTCTTCTACGATGTATTGGGTGACGTTGTCTGTGGTGGTTTCGCAATGCCTATTAGAGAAAATAAGGCCCAAGAAATCTATATAGTTTGCTCCGGTGAAATGATGGCAATGTACGCAGCCAACAACATTAGTAAGGGTATCGTCAAGTACGCTAACTCTGGTTCAGTTAGATTGGGTGGTTTGATCTGTAATTCTAGAAACACCGATAGAGAAGACGAATTGATCATCGCTTTGGCAAATAAGTTGGGTACTCAAATGATCCATTTCGTTCCAAGAGATAACGTAGTTCAAAGAGCTGAAATAAGAAGAATGACAGTTATAGAATACGATCCTAAAGCAAAGCAAGCCGACGAATACAGAGCCTTAGCTAGAAAGGTCGTAGATAATAAGTTGTTAGTTATCCCAAACCCTATTACCATGGATGAATTGGAAGAATTGTTAATGGAATTTGGTATTATGGAAGTAGAAGATGAATCAATCGTCGGTAAAACTGCTGAAGAAGTCTGA

**>yeast codon optimized *Azotobacter vinelandii* nifM (Genscript)**

ATGGCCTCAGAAAGATTAGCTGATGGTGACTCCAGATATTACTTGTTAAAAGTTGCCCATGAACAATTTGGTTGTGCTCCTGGTGAATTATCAGAAGAACAATTGCAACAAGCAGATAGAATTATAGGTAGACAAAGACACATAGAAGATGCAGTTTTAAGATCACCAGACGCCATAGGTGTTGTCATCCCACCTTCCCAATTGGAAGAAGCTTGGGCACATATTGCTTCAAGATATGAATCCCCTGAAGCCTTGCAACAAGCTTTAGATGCCCAAGCTTTGGACGCTGCTGGTATGAGAGCAATGTTGGCCAGAGAATTAAGAGTTGAAGCTGTCTTAGATTGTGTCTGCGCAGGTTTGCCAGAAATTAGTGATACAGACGTATCTTTGTACTACTTCAACCATGCTGAACAATTCAAGGTACCAGCACAACATAAAGCCAGACACATATTGGTTACTATAAATGAAGATTTTCCTGAAAACACAAGAGAAGCCGCTAGAACCAGAATCGAAACTATCTTGAAGAGATTGAGAGGTAAACCAGAAAGATTCGCTGAACAAGCAATGAAACACTCTGAATGTCCTACAGCTATGCAAGGTGGTTTGTTAGGTGAAGTAGTTCCAGGTACCTTGTATCCTGAATTAGATGCATGCTTGTTTCAAATGGCCAGAGGTGAATTATCACCAGTTTTGGAATCCCCTATTGGTTTCCATGTTTTATACTGTGAATCCGTCAGTCCAGCAAGACAATTGACCTTGGAAGAAATCTTGCCTAGATTGAGAGATAGATTGCAATTGAGACAAAGAAAGGCTTACCAAAGAAAGTGGTTGGAATCTTTGTTGCAACAAAATGCTACCTTGGAAAACTTAGCACATGGT

**>yeast codon optimized *Azotobacter vinelandii* nifD (Genscript)**

ATGACCGGATCCAGAGAAGAAGTTGAAAGTTTGATACAAGAAGTCTTGGAAGTTTATCCAGAAAAAGCTAGAAAGGACAGAAATAAGCATTTGGCAGTAAACGATCCTGCCGTCACCCAATCTAAAAAGTGTATCATCTCTAACAAAAAGTCACAACCAGGTTTAATGACTATTAGAGGTTGCGCCTATGCTGGTTCAAAAGGTGTTGTATGGGGTCCAATTAAGGATATGATACATATCTCCCACGGTCCTGTTGGTTGTGGTCAATACAGTAGAGCAGGTAGAAGAAACTACTACATAGGTACTACAGGTGTTAATGCCTTCGTAACTATGAACTTCACATCAGATTTCCAAGAAAAGGACATCGTTTTTGGTGGTGACAAAAAGTTGGCTAAGTTGATCGAcGAAGTAGAAACATTGTTCCCATTGAACAAAGGTATCTCCGTCCAAAGTGAATGCCCTATTGGTTTGATAGGTGACGACATTGAATCCGTAAGTAAAGTCAAGGGTGCAGAATTATCCAAGACCATAGTTCCAGTAAGATGTGAAGGTTTCAGAGGTGTTTCCCAAAGTTTGGGTCATCACATTGCAAATGATGCCGTCAGAGACTGGGTTTTAGGTAAAAGAGATGAAGACACCACTTTTGCATCTACTCCTTATGATGTTGCCATCATTGGTGACTACAACATTGGTGGTGACGCTTGGTCTTCAAGAATCTTGTTGGAAGAAATGGGTTTGAGATGTGTTGCTCAATGGTCTGGTGACGGTTCCATCTCAGAAATAGAATTGACACCAAAGGTTAAGTTGAATTTGGTACATTGCTACAGATCTATGAACTACATCTCAAGACACATGGAAGAAAAGTACGGTATCCCATGGATGGAATACAATTTCTTTGGTCCTACAAAAACCATTGAATCTTTGAGAGCCATAGCTGCAAAGTTCGATGAATCAATCCAAAAGAAATGTGAAGAAGTTATCGCAAAGTATAAGCCAGAATGGGAAGCTGTCGTTGCAAAATACAGACCTAGATTGGAAGGTAAAAGAGTTATGTTGTACATCGGTGGTTTAAGACCTAGACATGTAATTGGTGCTTACGAAGATTTGGGTATGGAAGTAGTCGGTACTGGTTATGAATTCGCACATAATGATGACTACGACAGAACAATGAAAGAAATGGGTGACTCCACCTTGTTATACGATGACGTCACTGGTTACGAATTCGAAGAATTCGTTAAGAGAATTAAGCCAGATTTGATCGGTTCTGGTATCAAAGAAAAGTTTATCTTCCAAAAGATGGGTATTCCTTTTAGACAAATGCATTCCTGGGATTATAGTGGTCCTTACCACGGTTTTGATGGTTTCGCCATTTTTGCTAGAGATATGGACATGACTTTGAATAACCCTTGTTGGAAAAAGTTACAAGCCCCTTGGGAAGCCAGTGAAGGTGCCGAAAAAGTTGCCGCATCCGCCTGA

**>yeast codon optimized *Azotobacter vinelandii* nifK (Genscript)**

ATGTCTCAACAAGTTGATAAGATTAAAGCATCTTACCCATTGTTTTTAGATCAAGACTACAAGGATATGTTAGCCAAAAAGAGAGACGGTTTCGAAGAAAAGTACCCTCAAGATAAGATCGACGAAGTTTTTCAATGGACTACAACCAAGGAATACCAAGAATTGAACTTCCAAAGAGAAGCATTGACTGTAAACCCAGCAAAAGCCTGTCAACCTTTGGGTGCAGTCTTGTGCGCCTTAGGTTTTGAAAAGACAATGCCTTATGTCCATGGTTCACAAGGTTGTGTTGCCTACTTCAGATCCTACTTCAACAGACACTTCAGAGAACCTGTCTCTTGTGTTTCTGATTCAATGACCGAAGACGCTGCAGTTTTCGGTGGTCAACAAAACATGAAGGATGGTTTGCAAAACTGTAAAGCTACTTACAAGCCAGACATGATCGCTGTATCAACTACATGCATGGCAGAAGTCATTGGTGACGACTTGAACGCTTTCATAAACAACTCCAAAAAGGAAGGTTTTATTCCAGATGAATTCCCAGTTCCTTTTGCACATACTCCTTCATTTGTAGGTTCCCACGTCACAGGTTGGGATAACATGTTCGAAGGTATAGCAAGATACTTCACATTGAAGAGTATGGATGACAAGGTTGTAGGTTCTAATAAGAAAATTAATATCGTTCCAGGTTTCGAAACTTATTTGGGTAACTTCAGAGTCATAAAAAGAATGTTGTCAGAAATGGGTGTTGGTTACTCATTGTTATCCGATCCAGAAGAAGTTTTGGATACACCTGCCGACGGTCAATTCAGAATGTACGCTGGTGGTACCACTCAAGAAGAAATGAAAGATGCTCCAAATGCATTAAACACTGTATTGTTACAACCTTGGCATTTGGAAAAGACTAAGAAATTCGTTGAAGGTACCTGGAAACACGAAGTACCAAAGTTAAACATTCCTATGGGTTTGGATTGGACAGACGAATTCTTGATGAAAGTAAGTGAAATCTCTGGTCAACCAATCCCTGCCTCATTAACCAAGGAAAGAGGTAGATTGGTCGATATGATGACCGACTCCCATACTTGGTTACACGGTAAAAGATTTGCTTTGTGGGGTGACCCAGACTTCGTTATGGGTTTGGTAAAGTTCTTGTTGGAATTGGGTTGTGAACCTGTCCATATATTGTGCCACAACGGTAACAAAAGATGGAAAAAGGCCGTTGATGCTATTTTGGCCGCTTCTCCTTATGGTAAAAATGCTACCGTTTACATTGGTAAAGATTTGTGGCATTTGAGAAGTTTGGTATTCACTGATAAACCTGACTTTATGATCGGTAACTCTTACGGTAAATTCATTCAAAGAGATACTTTGCATAAGGGTAAAGAATTCGAAGTTCCATTGATCAGAATTGGTTTTCCTATTTTCGATAGACATCACTTACATAGAAGTACAACCTTGGGTTACGAAGGTGCAATGCAAATCTTGACTACATTGGTTAACTCTATCTTAGAAAGATTGGATGAAGAAACCAGAGGTATGCAAGCAACAGATTACAACCACGACTTAGTTAGATGA

**>yeast codon optimized *Azotobacter vinelandii* nifV (Genscript)**

ATGGCAAGTGTAATAATAGACGATACCACATTGAGAGACGGTGAACAAAGTGCAGGTGTAGCATTCAACGCAGACGAAAAGATAGCCATCGCCAGAGCTTTGGCAGAATTAGGTGTCCCAGAATTAGAAATTGGTATACCTTCTATGGGTGAAGAAGAAAGAGAAGTAATGCATGCCATTGCTGGTTTGGGTTTATCTTCAAGATTGTTAGCTTGGTGTAGATTATGCGATGTTGACTTGGCTGCAGCCAGATCAACAGGTGTTACCATGGTAGATTTGTCTTTACCAGTTTCAGACTTGATGTTGCATCACAAATTGAACAGAGATAGAGACTGGGCTTTAAGAGAAGTAGCAAGATTGGTCGGTGAAGCTAGAATGGCAGGTTTGGAAGTTTGTTTAGGTTGCGAAGATGCCTCCAGAGCTGACTTGGAATTTGTTGTACAAGTCGGTGAAGTTGCCCAAGCTGCAGGTGCTAGAAGATTAAGATTTGCTGATACTGTAGGTGTCATGGAACCTTTCGGCATGTTGGATAGATTCAGATTTTTGTCTAGAAGATTGGATATGGAATTGGAAGTTCATGCTCACGATGACTTCGGTTTAGCAACTGCCAACACATTGGCCGCTGTTATGGGTGGTGCAACACATATCAACACTACAGTAAACGGTTTAGGTGAAAGAGCTGGTAATGCAGCCTTGGAAGAATGTGTTTTGGCCTTAAAGAACTTACATGGTATAGATACCGGTATCGACACTAGAGGTATCCCAGCAATTTCTGCCTTGGTTGAAAGAGCATCAGGTAGACAAGTAGCCTGGCAAAAATCAGTCGTTGGTGCCGGTGTCTTTACACATGAAGCTGGTATTCACGTTGATGGTTTGTTGAAGCATAGAAGAAACTACGAAGGTTTGAACCCTGATGAATTGGGTAGATCCCACAGTTTGGTATTAGGTAAACATTCCGGTGCTCACATGGTCAGAAATACCTACAGAGATTTGGGTATTGAATTGGCTGACTGGCAAAGTCAAGCATTGTTAGGTAGAATAAGAGCTTTTTCCACCAGAACTAAGAGAAGTCCACAACCTGCAGAATTGCAAGATTTCTACAGACAATTGTGCGAACAAGGTAACCCAGAATTGGCTGCAGGTGGTATGGCATAA

**>yeast codon optimized *Azotobacter vinelandii* nifX (Genscript)**

ATGAGTTCTCCAACAAGACAGTTACAGGTTTTAGATAGTGAGGATGATGGTACATTATTGAAGGTGGCATTTGCTAGTTCTGATAGAGAACTTGTTGATCAACATTTTGGATCTTCAAGATCTTTCGCTATCTATGGTGTGAATCCTGAAAGGAGTCAACTTTTGTCTGTTGTGGAGTTTGGAGAATTAGAGCAGGATGGTAACGAGGATAAGCTTGCTAGAAAAATAGATCTCTTAGATGGATGTGTTGCTGTGTACTGTTGCGCATGCGGTGCTTCAGCAGTTAGACAATTGATGGCAATAGGAGTTCAGCCAATTAAGGTGAGTGAAGGTGCTAGAATTGCAGAACTTATCGAGGCTTTGCAAGTTGAACTCAGGGAGGGACCTTCAGCATGGCTCGCTAAAGCAATTCAGAGAACTAGGGGACCAGATATGAGAAGGTTCGATGCTATGGCTGCAGAAGGTTGGGATGAGTGA

**>Arabidopsis codon optimized *Azotobacter vinelandii* nifF (Genscript)**

ATGGCTAAGATTGGATTATTCTTCGGTAGTAACACAGGAAAAACAAGGAAGGTGGCTAAAAGTATTAAAAAGAGATTTGATGATGAAACAATGTCTGATGCTTTGAATGTTAACAGAGTGTCAGCTGAAGATTTCGCACAATACCAGTTCCTTATATTGGGAACTCCTACACTCGGAGAAGGAGAGTTACCAGGTCTTTCTTCAGATTGTGAAAATGAGAGTTGGGAAGAGTTTCTCCCTAAGATCGAGGGATTAGATTTTTCTGGAAAGACTGTTGCTTTGTTCGGACTCGGAGATCAAGTGGGATATCCAGAAAACTACTTAGATGCTCTTGGAGAGTTGTATTCTTTCTTTAAGGATAGAGGAGCAAAAATAGTTGGTTCATGGAGTACCGATGGATACGAATTTGAGAGTTCTGAAGCTGTTGTGGATGGAAAGTTCGTTGGTCTCGCACTCGATCTTGATAACCAATCAGGAAAAACTGATGAAAGGGTGGCTGCATGGCTTGCTCAGATCGCACCTGAGTTCGGTTTGAGTCTCTGA

**>yeast codon optimized *Azotobacter vinelandii* nifQ (Genscript)**

ATGGGTTCCGCTGCCGCACACAGAGGTGACACAACACAAGCCGTTAGACATGACAGAGCAAATCACTTATGGTTAGAAAGAATAGTCAGATCTCAAAGAGATGGTTTATCATGTTTGCCATTTCATTTGGGTTTGGACGAAAGATCCTATGCCGAATTAATTAGAACTCACTTCCCTGAATTGGCTGGTCAAACATCTGCATCATTAGGTTCCTTGGCTCATGAATGCAGTGAATTGAGAGAAGATTTGTTGGAAATGAGAAGAGACGAATGGGAAGAATTGAGAGTCTTGTTATTGGATGGTAGAAGAGGTGACGACCCAGAAGAATTATGGATGGCATCTATAGTTGCTGCAGCCTGTTTAGGTGGTGACCATTTGTGGAGAGACTTAGGTTTGGAATCAAGAGAAACCTTGAGAGTTTTGTTGATGCATAACTTCCCTCACTTGGCCGAAAGAAACGTTAAAAATATGAGATGGAAAAAGTTTTTCTATAAGCAATTGTGTGAACAAGATGGTGGTTACGTATGCAGATCCCCAAGTTGTGAACAATGCCCTTCTCATCACGATTGTTTCGGTGCAGAAATTTGA

**>Arabidopsis codon optimized *Klebsiella pneumoniae* nifJ (Genscript)**

ATGTCAGGAAAGATGAAGACTATGGATGGAAACGCTGCTGCTGCTTGGATCAGTTACGCATTCACCGAGGTTGCTGCTATCTACCCAATCACCCCTTCTACTCCAATGGCTGAAAATGTTGATGAGTGGGCTGCACAAGGAAAGAAAAACTTATTTGGTCAGCCTGTTAGACTTATGGAAATGCAAAGTGAGGCTGGAGCTGCAGGTGCAGTGCATGGAGCTTTGCAGGCTGGTGCACTCACTACAACCTATACTGCTTCTCAAGGTCTTTTGCTCATGATACCTAACATGTACAAAATCGCTGGAGAATTACTTCCAGGTGTTTTTCATGTGTCAGCTAGGGCATTAGCTACCAATAGTCTTAACATTTTCGGAGATCACCAAGATGTTATGGCTGTGAGACAGACTGGTTGTGCAATGCTTGCTGAGAATAACGTTCAACAAGTTATGGATCTTTCAGCAGTTGCTCATCTCGCTGCAATCAAAGGAAGGATACCTTTTGTGAATTTCTTTGATGGTTTCAGAACTTCTCACGAAATTCAGAAGATCGAGGTTTTGGAATATGAGCAACTCGCAACATTGCTCGATAGGCCAGCTTTGGATTCTTTTAGAAGGAACGCTCTCCATCCTGATCACCCAGTTATTAGAGGAACAGCTCAAAATCCTGATATCTACTTTCAGGAAAGGGAGGCTGGTAACAGATTCTATCAGGCTTTGCCAGATATTGTTGAGTCTTACATGACTCAAATATCAGCTTTGACAGGAAGGGAATATCATCTCTTCAATTACACTGGTGCTGCAGATGCAGAGAGAGTTATTATCGCTATGGGTTCTGTTTGTGATACAGTGCAGGAGGTTGTGGATACCCTTAACGCTGCAGGAGAAAAAGTTGGTTTACTTTCTGTGCATCTTTTTAGACCTTTCTCACTTGCACACTTTTTCGCTCAACTTCCAAAAACTGTTCAGAGGATTGCTGTGTTGGATAGAACAAAGGAACCTGGAGCACAAGCTGAGCCACTCTGCCTTGATGTTAAGAACGCATTCTATCATCACGATGATGCTCCTTTGATCGTGGGAGGTAGATACGCTTTGGGTGGTAAAGATGTTCTCCCAAACGATATAGCTGCAGTGTTCGATAATCTTAACAAGCCTCTTCCTATGGATGGATTCACTCTTGGTATCGTTGATGATGTGACTTTCACAAGTTTACCTCCAAGACAACAGACACTTGCAGTTTCTCATGATGGTATCACCGCTTGTAAATTTTGGGGAATGGGTAGTGATGGAACCGTGGGTGCAAATAAGTCTGCTATTAAGATTATTGGAGATAAGACTCCTCTTTATGCACAAGCTTACTTCAGTTACGATTCTAAGAAATCAGGAGGTATTACTGTTTCACATCTTAGATTTGGAGATAGGCCTATTAATAGTCCATACTTGATTCACAGGGCTGATTTCATCTCATGTAGTCAACAGTCTTATGTTGAAAGATACGATTTGCTCGATGGACTTAAACCTGGAGGTACATTTTTACTTAACTGCTCTTGGTCAGATGCTGAATTGGAGCAGCATCTCCCAGTTGGTTTTAAGAGATATCTCGCTAGGGAAAATATACACTTCTACACTCTTAACGCAGTGGATATTGCTAGGGAGTTGGGACTCGGAGGTAGATTCAATATGCTCATGCAAGCTGCATTTTTCAAGCTTGCTGCAATCATAGATCCTCAGACAGCTGCAGATTATTTGAAGCAAGCTGTTGAGAAAAGTTACGGATCTAAGGGTGCTGCAGTGATTGAGATGAATCAGAGAGCAATCGAGTTGGGTATGGCTTCTCTTCATCAAGTTACTATCCCTGCACACTGGGCTACACTTGATGAACCAGCTGCACAGGCATCAGCTATGATGCCTGATTTCATTAGGGATATATTGCAACCTATGAATAGACAATGTGGAGATCAGCTCCCTGTTTCTGCTTTTGTGGGTATGGAAGATGGTACATTCCCATCAGGAACCGCTGCATGGGAAAAGAGAGGTATTGCTCTTGAGGTTCCTGTGTGGCAACCAGAAGGATGTACTCAATGCAATCAGTGTGCTTTCATATGCCCTCATGCTGCAATTAGACCAGCTTTGCTCAACGGTGAAGAGCACGATGCTGCACCTGTTGGATTACTTTCAAAACCAGCACAGGGTGCTAAGGAATATCATTACCACTTGGCTATATCACCTCTCGATTGCAGTGGATGTGGTAATTGCGTTGATATTTGTCCAGCAAGGGGAAAGGCTCTCAAGATGCAATCACTTGATAGTCAAAGACAGATGGCTCCTGTTTGGGATTATGCATTAGCTCTTACACCTAAGTCTAACCCTTTTAGAAAGACTACAGTGAAAGGATCTCAGTTTGAAACCCCTTTGCTCGAGTTCTCAGGTGCATGTGCTGGATGCGGAGAGACCCCATATGCTAGGTTAATCACTCAACTTTTCGGAGATAGAATGTTGATAGCAAATGCTACAGGATGCTCTTCAATCTGGGGTGCATCTGCTCCTTCAATACCATACACCACTAACCATAGAGGACACGGTCCTGCATGGGCTAATTCACTTTTTGAAGATAACGCTGAGTTCGGATTGGGTATGATGCTCGGAGGTCAAGCAGTTAGACAACAGATCGCTGATGATATGACAGCTGCATTGGCTCTCCCAGTGAGTGATGAATTGTCTGATGCAATGAGACAATGGCTCGCTAAACAGGATGAAGGAGAGGGTACTAGAGAGAGGGCTGATAGATTATCTGAAAGGCTTGCTGCAGAAAAGGAGGGAGTTCCTTTACTTGAGCAATTGTGGCAGAACAGAGATTATTTTGTGAGAAGGTCACAGTGGATTTTCGGAGGAGATGGATGGGCTTACGATATCGGTTTTGGAGGTTTGGATCATGTTCTCGCTTCTGGAGAGGATGTTAATATCCTTGTGTTCGATACCGAAGTTTATTCAAACACTGGTGGTCAGAGTTCTAAAAGTACACCAGTGGCTGCAATTGCTAAATTTGCTGCACAAGGAAAGAGAACCAGGAAGAAAGATTTGGGAATGATGGCTATGTCTTATGGTAATGTTTACGTGGCACAGGTTGCTATGGGAGCAGATAAGGATCAAACTCTTAGAGCAATTGCTGAAGCAGAGGCTTGGCCTGGTCCATCACTCGTGATCGCTTACGCTGCATGTATTAATCATGGACTTAAAGCTGGTATGAGATGCAGTCAGAGGGAAGCTAAGAGAGCAGTTGAGGCTGGATATTGGCATTTGTGGAGGTACCACCCTCAAAGAGAAGCTGAGGGAAAGACTCCTTTCATGCTCGATAGTGAAGAGCCAGAAGAGTCTTTTAGGGATTTCTTGCTCGGTGAAGTTAGATATGCTAGTTTGCATAAGACAACCCCACACTTAGCAGATGCTCTTTTCTCTAGAACTGAAGAGGATGCAAGAGCTAGGTTCGCACAATACAGAAGGCTTGCTGGAGAAGAGTGA

**>TDH2t:** terminator of *S. cerevisiae* YJR009C (136 bp)

TAAATTTAACTCCTTAAGTTACTTTAATGATTTAGTTTTTATTATTAATAATTCATGCTCATGACATCTCATATACACGTTTATAAAACTTAAATAGATTGAAAATGTATTAAAGATTCCTCAGGGATTGGATTTT

>**Cyc1t:** terminator of *S. cerevisiae* YJR048W (190 bp)

ATCCGCTCTAACCGAAAAGGAAGGAGTTAGACAACCTGAAGTCTAGGTCCCTATTTATTTTTTTATAGTTATGTTAGTATTAAGAACGTTATTTATATTTCAAATTTTTCTTTTTTTTCTGTACAGACGCGTGTACGCATGTAACATTATACTGAAAACCTTGCTTGAGAAGGTTTTGGGACGCTCGAAG

**>ADH2t:** terminator of S. cerevisiae YMR303C (376 bp)

GATTTATAGTTTTCATTATCAAGTATGCCTATATTAGTATATAGCATCTTTAGATGACAGTGTTCGAAGTTTCACGAATAAAAGATAATATTCTACTTTTTGCTCCCACCGCGTTTGCTAGCACGAGTGAACACCATCCCTCGCCTGTGAGTTGTACCCATTCCTCTAAACTGTAGACATGGTAGCTTCAGCAGTGTTCGTTATGTACGGCATCCTCCAACAAACAGTCGGTTATAGTTTGTCCTGCTCCTCTGAATCGTCTCCCTCGATATTTCTCATTTTCCTTCGCATGCCAGCATTGAAATGATCGAAGTTCAATGATGAAACGGTAATTCTTCTGTCATTTACTCATCTCATCTCATCAAGTTATATAATT

**>loxP-P_AgTEF_-kanMX-T_AgTEF_-loxP:** from pUG6 (P30114)

ATAACTTCGTATAATGTATGCTATACGAAGTTATTAGGTCTAGAGATCTGTTTAGCTTGCCTCGTCCCCGCCGGGTCACCCGGCCAGCGACATGGAGGCCCAGAATACCCTCCTTGACAGTCTTGACGTGCGCAGCTCAGGGGCATGATGTGACTGTCGCCCGTACATTTAGCCCATACATCCCCATGTATAATCATTTGCATCCATACATTTTGATGGCCGCACGGCGCGAAGCAAAAATTACGGCTCCTCGCTGCAGACCTGCGAGCAGGGAAACGCTCCCCTCACAGACGCGTTGAATTGTCCCCACGCCGCGCCCCTGTAGAGAAATATAAAAGGTTAGGATTTGCCACTGAGGTTCTTCTTTCATATACTTCCTTTTAAAATCTTGCTAGGATACAGTTCTCACATCACATCCGAACATAAACAACCATGGGTAAGGAAAAGACTCACGTTTCGAGGCCGCGATTAAATTCCAACATGGATGCTGATTTATATGGGTATAAATGGGCTCGCGATAATGTCGGGCAATCAGGTGCGACAATCTATCGATTGTATGGGAAGCCCGATGCGCCAGAGTTGTTTCTGAAACATGGCAAAGGTAGCGTTGCCAATGATGTTACAGATGAGATGGTCAGACTAAACTGGCTGACGGAATTTATGCCTCTTCCGACCATCAAGCATTTTATCCGTACTCCTGATGATGCATGGTTACTCACCACTGCGATCCCCGGCAAAACAGCATTCCAGGTATTAGAAGAATATCCTGATTCAGGTGAAAATATTGTTGATGCGCTGGCAGTGTTCCTGCGCCGGTTGCATTCGATTCCTGTTTGTAATTGTCCTTTTAACAGCGATCGCGTATTTCGTCTCGCTCAGGCGCAATCACGAATGAATAACGGTTTGGTTGATGCGAGTGATTTTGATGACGAGCGTAATGGCTGGCCTGTTGAACAAGTCTGGAAAGAAATGCATAAGCTTTTGCCATTCTCACCGGATTCAGTCGTCACTCATGGTGATTTCTCACTTGATAACCTTATTTTTGACGAGGGGAAATTAATAGGTTGTATTGATGTTGGACGAGTCGGAATCGCAGACCGATACCAGGATCTTGCCATCCTATGGAACTGCCTCGGTGAGTTTTCTCCTTCATTACAGAAACGGCTTTTTCAAAAATATGGTATTGATAATCCTGATATGAATAAATTGCAGTTTCATTTGATGCTCGATGAGTTTTTCTAATCAGTACTGACAATAAAAAGATTCTTGTTTTCAAGAACTTGTCATTTGTATAGTTTTTTTATATTGTAGTTGTTCTATTTTAATCAAATGTTAGCGTGATTTATATTTTTTTTCGCCTCGACATCATCTGCCCAGATGCGAAGTTAAGTGCGCAGAAAGTAATATCATGCGTCAATCGTATGTGAATGCTGGTCGCTATACTGCTGTCGATTCGATACTAACGCCGCCATCCAGTGTCGAAAACGAGCTCTCGAGAACCCTTAATATAACTTCGTATAATGTATGCTATACGAAGTTA

**>loxP-P_AgTEF_-hph-T_AgTEF_-loxP:** from pUG75 (P30671)

ATAACTTCGTATAATGTATGCTATACGAAGTTATTAGGTCTAGAGATCTGTTTAGCTTGCCTCGTCCCCGCCGGGTCACCCGGCCAGCGACATGGAGGCCCAGAATACCCTCCTTGACAGTCTTGACGTGCGCAGCTCAGGGGCATGATGTGACTGTCGCCCGTACATTTAGCCCATACATCCCCATGTATAATCATTTGCATCCATACATTTTGATGGCCGCACGGCGCGAAGCAAAAATTACGGCTCCTCGCTGCAGACCTGCGAGCAGGGAAACGCTCCCCTCACAGACGCGTTGAATTGTCCCCACGCCGCGCCCCTGTAGAGAAATATAAAAGGTTAGGATTTGCCACTGAGGTTCTTCTTTCATATACTTCCTTTTAAAATCTTGCTAGGATACAGTTCTCACATCACATCCGAACATAAACAACCATGGGTAAAAAGCCTGAACTCACCGCGACGTCTGTCGAGAAGTTTCTGATCGAAAAGTTCGACAGCGTCTCCGACCTGATGCAGCTCTCGGAGGGCGAAGAATCTCGTGCTTTCAGCTTCGATGTAGGAGGGCGTGGATATGTCCTGCGGGTAAATAGCTGCGCCGATGGTTTCTACAAAGATCGTTATGTTTATCGGCACTTTGCATCGGCCGCGCTCCCGATTCCGGAAGTGCTTGACATTGGGGAATTCAGCGAGAGCCTGACCTATTGCATCTCCCGCCGTGCACAGGGTGTCACGTTGCAAGACCTGCCTGAAACCGAACTGCCCGCTGTTCTGCAGCCGGTCGCGGAGGCAATGGATGCGATCGCTGCGGCCGATCTTAGCCAGACGAGCGGGTTCGGCCCATTCGGACCGCAAGGAATCGGTCAATACACTACATGGCGTGATTTCATATGCGCGATTGCTGATCCCCATGTGTATCACTGGCAAACTGTGATGGACGACACCGTCAGTGCGTCCGTCGCGCAGGCTCTCGATGAGCTGATGCTTTGGGCCGAGGACTGCCCCGAAGTCCGGCACCTCGTGCACGCGGATTTCGGCTCCAACAATGTCCTGACGGACAATGGCCGCATAACAGCGGTCATTGACTGGAGCGAGGCGATGTTCGGGGATTCCCAATACGAGGTCGCCAACATCTTCTTCTGGAGGCCGTGGTTGGCTTGTATGGAGCAGCAGACGCGCTACTTCGAGCGGAGGCATCCGGAGCTTGCAGGATCGCCGCGGCTCCGGGCGTATATGCTCCGCATTGGTCTTGACCAACTCTATCAGAGCTTGGTTGACGGCAATTTCGATGATGCAGCTTGGGCGCAGGGTCGATGCGACGCAATCGTCCGATCCGGAGCCGGGACTGTCGGGCGTACACAAATCGCCCGCAGAAGCGCGGCCGTCTGGACCGATGGCTGTGTAGAAGTACTCGCCGATAGTGGAAACCGACGCCCCAGCACTCGTCCGAGGGCAAAGGAATAATCAGTACTGACAATAAAAAGATTCTTGTTTTCAAGAACTTGTCATTTGTATAGTTTTTTTATATTGTAGTTGTTCTATTTTAATCAAATGTTAGCGTGATTTATATTTTTTTTCGCCTCGACATCATCTGCCCAGATGCGAAGTTAAGTGCGCAGAAAGTAATATCATGCGTCAATCGTATGTGAATGCTGGTCGCTATACTGCTGTCGATTCGATACTAACGCCGCCATCCAGTGTCGAAAACGAGCTCTCGAGAACCCTTAATATAACTTCGTATAATGTATGCTATACGAAGTTAT
