# Supplementary material for: Adaptation of the GoldenBraid modular cloning system and creation of a toolkit for the expression of heterologous proteins in yeast mitochondria
Source: BMC Biotechnol. 2017 Nov 13;17:80. doi: 10.1186/s12896-017-0393-y (PMC5683533; doi:10.1186/s12896-017-0393-y)
Supplement: Supplementary file 4 — (.doc) Results of BLAST (yeastgenome.org/blast-sgd; matrix BLOSUM62) of the AOH sequences cloned in the adapted vectors and yeast strain W303 AND CEN-PK genomes. (DOCX 26 kb) [file 12896_2017_393_MOESM4_ESM.docx]

**Adaptation of the GoldenBraid modular cloning system and creation of a toolkit for the expression of mitochondrial proteins in yeast.** Ana Pérez-González, Ryan Kniewel, Marcel Veldhuizen, Hemant K. Verma, Mónica Navarro-Rodríguez, Luis M. Rubio and Elena Caro.

**Figure S2**

*Saccharomyces cerevisiae* strain W303

5’-YPRCΔ15

(914 basepairs)

Database: W303_MPG_2012_ALAV00000000.fsa

Score = 681.2 bits (4500), Expect = 3.9e-198, P = 3.9e-198
Identities = 904/914 (98%), Frame = +1 / +1

Query: 1 CGAGATAGTTTGACGTTCGTTTTTTACTTTGAATATACTCGTAGTCTTTTTACTTTTTGA 60

||||||||||||||||||||||||||||||||||||||||||||||||||||||||||||

Sbjct: 333250 CGAGATAGTTTGACGTTCGTTTTTTACTTTGAATATACTCGTAGTCTTTTTACTTTTTGA 333309

Query: 61 GTATNNNNNNNNNNTGACTAGCAAAATAAAATTAGTAGTCTAAAAAAGAAAGCTCGCACT 120

|||| ||||||||||||||||||||||||||||||||||||||||||||||

Sbjct: 333310 GTATAAAAAAAAAATGACTAGCAAAATAAAATTAGTAGTCTAAAAAAGAAAGCTCGCACT 333369

Query: 121 CAGGATCGAACTAAGGACCAACAGATTTGCAATCTGCTGCGCTACCACTGCGCCATACGA 180

||||||||||||||||||||||||||||||||||||||||||||||||||||||||||||

Sbjct: 333370 CAGGATCGAACTAAGGACCAACAGATTTGCAATCTGCTGCGCTACCACTGCGCCATACGA 333429

Query: 181 GCTTTTGAATTATGGTAATTTTGATTATCCTAGAATGTTATATCTCAATATCTCAATATA 240

||||||||||||||||||||||||||||||||||||||||||||||||||||||||||||

Sbjct: 333430 GCTTTTGAATTATGGTAATTTTGATTATCCTAGAATGTTATATCTCAATATCTCAATATA 333489

Query: 241 TTTTGGACATCTATGAAACACCCATAAAGCAGCCGCTACCAAACAGACAAGATTCAGTAT 300

||||||||||||||||||||||||||||||||||||||||||||||||||||||||||||

Sbjct: 333490 TTTTGGACATCTATGAAACACCCATAAAGCAGCCGCTACCAAACAGACAAGATTCAGTAT 333549

Query: 301 GTAAGGTAAATACCTTTTTGCACAGTTAAACTACCCAAACTTATTAAAGCTTGATAAATT 360

||||||||||||||||||||||||||||||||||||||||||||||||||||||||||||

Sbjct: 333550 GTAAGGTAAATACCTTTTTGCACAGTTAAACTACCCAAACTTATTAAAGCTTGATAAATT 333609

Query: 361 ACTGAAATTCCACCTTTCAGTTAGATTCAGGCCTCATATAGATTAGATATAGGGTACGTA 420

||||||||||||||||||||||||||||||||||||||||||||||||||||||||||||

Sbjct: 333610 ACTGAAATTCCACCTTTCAGTTAGATTCAGGCCTCATATAGATTAGATATAGGGTACGTA 333669

Query: 421 ACATTCTGTCAACCAAGTTGTTGGAATGAAAGTCTAAAATGTCATCTATTCGGTAGCACT 480

||||||||||||||||||||||||||||||||||||||||||||||||||||||||||||

Sbjct: 333670 ACATTCTGTCAACCAAGTTGTTGGAATGAAAGTCTAAAATGTCATCTATTCGGTAGCACT 333729

Query: 481 CATGTTACTAGTATACTGTCACATGCGGTGTAACGTGGGGACATAAAACAGACATCAAAT 540

||||||||||||||||||||||||||||||||||||||||||||||||||||||||||||

Sbjct: 333730 CATGTTACTAGTATACTGTCACATGCGGTGTAACGTGGGGACATAAAACAGACATCAAAT 333789

Query: 541 ATAATGGAAGCTGAAATGCAAAGATCGATAATGTAATAGGAATGAAACATATAAAACGAA 600

||||||||||||||||||||||||||||||||||||||||||||||||||||||||||||

Sbjct: 333790 ATAATGGAAGCTGAAATGCAAAGATCGATAATGTAATAGGAATGAAACATATAAAACGAA 333849

Query: 601 AGGAGAAGTAATGGTAATATTAGTATGTAGAAATACCGATTCAATTTTGGGGATTCTTAT 660

||||||||||||||||||||||||||||||||||||||||||||||||||||||||||||

Sbjct: 333850 AGGAGAAGTAATGGTAATATTAGTATGTAGAAATACCGATTCAATTTTGGGGATTCTTAT 333909

Query: 661 ATTCTCGAGAGAATTTCTAGTATAATCTGTATACATAATATTATAGGCTTTACCAACAAT 720

||||||||||||||||||||||||||||||||||||||||||||||||||||||||||||

Sbjct: 333910 ATTCTCGAGAGAATTTCTAGTATAATCTGTATACATAATATTATAGGCTTTACCAACAAT 333969

Query: 721 GGAATTTCGACAATTATCATATTATTCACCAATTAATCACAAGTTGGTAATGAGTTTGAT 780

||||||||||||||||||||||||||||||||||||||||||||||||||||||||||||

Sbjct: 333970 GGAATTTCGACAATTATCATATTATTCACCAATTAATCACAAGTTGGTAATGAGTTTGAT 334029

Query: 781 AACAAGTTACTTTCTTAACAACGTTAGTATCGTCAAAACACTCGGTTTTACTCGAGCTTG 840

||||||||||||||||||||||||||||||||||||||||||||||||||||||||||||

Sbjct: 334030 AACAAGTTACTTTCTTAACAACGTTAGTATCGTCAAAACACTCGGTTTTACTCGAGCTTG 334089

Query: 841 TAGCACAATAATACCGTGTAGAGTTCTGTATTGTTCTTCTTAGTGCTTGTATATGCTCAT 900

||||||||||||||||||||||||||||||||||||||||||||||||||||||||||||

Sbjct: 334090 TAGCACAATAATACCGTGTAGAGTTCTGTATTGTTCTTCTTAGTGCTTGTATATGCTCAT 334149

Query: 901 CCCGACCTTCCATT 914

||||||||||||||

Sbjct: 334150 CCCGACCTTCCATT 334163

3’-YPRCΔ15

(993 basepairs)

Database: W303_MPG_2012_ALAV00000000.fsa

Score = 685.9 bits (4531), Expect = 1.6e-199, P = 1.6e-199
Identities = 931/993 (93%), Frame = +1 / +1

Query: 1 TTTGCGAAACCCTATGCTCTGTTGTTCGGATTTGAAATTTTAAAACTACATTAATGTGTT 60

||||||||||||||||||||||||||||||||||||||||||||||||||||||||||||

Sbjct: 334466 TTTGCGAAACCCTATGCTCTGTTGTTCGGATTTGAAATTTTAAAACTACATTAATGTGTT 334525

Query: 61 AGTTTTTCTTTCTTTCTTTCTTTGTCTTGACGTGATTTGGACTTCTGTCTTGCATTCGCG 120

||||||||||||||||||||||||||||||||||||||||||||||||||||||||||||

Sbjct: 334526 AGTTTTTCTTTCTTTCTTTCTTTGTCTTGACGTGATTTGGACTTCTGTCTTGCATTCGCG 334585

Query: 121 TCCATTCATCTGACCCAATATTCCTTTTGGTTTTGTTATCCTTATAAAAAGAAAGGAAGC 180

||||||||||||||||||||||||||||||||||||||||||||||||||||||||||||

Sbjct: 334586 TCCATTCATCTGACCCAATATTCCTTTTGGTTTTGTTATCCTTATAAAAAGAAAGGAAGC 334645

Query: 181 TTCTTAGAGGGANNNNNNNNNNNNNNNNNNNNNNNNNNNNNNNNNNNNNNNNNNNNNNNN 240

||||||||||||

Sbjct: 334646 TTCTTAGAGGGAAAAAAATGATGAAGAGTAATGCCAAAATATAAATAAATAAATAAATAT 334705

Query: 241 NNNNNNCATTTTCTATTTTTAATAGAATAAGAAGAGCATCTTAAGATTACAATTTCAAGA 300

||||||||||||||||||||||||||||||||||||||||||||||||||||||

Sbjct: 334706 GAAAATCATTTTCTATTTTTAATAGAATAAGAAGAGCATCTTAAGATTACAATTTCAAGA 334765

Query: 301 AATAGTTTACACAGTATATCCAATAACTCCAATAAACTACTTTCCTATACAAATTTCTAT 360

||||||||||||||||||||||||||||||||||||||||||||||||||||||||||||

Sbjct: 334766 AATAGTTTACACAGTATATCCAATAACTCCAATAAACTACTTTCCTATACAAATTTCTAT 334825

Query: 361 GGTGGGATTAATAGTAAAACTTCTGTACTTCTCTAATTCACCAAGAAATTAAGGTAAACA 420

||||||||||||||||||||||||||||||||||||||||||||||||||||||||||||

Sbjct: 334826 GGTGGGATTAATAGTAAAACTTCTGTACTTCTCTAATTCACCAAGAAATTAAGGTAAACA 334885

Query: 421 TCTGGTAAGCACTATCCAGCTTTTTGCTATTACACATATGGCTTTTCTGCAATCATTTCT 480

||||||||||||||||||||||||||||||||||||||||||||||||||||||||||||

Sbjct: 334886 TCTGGTAAGCACTATCCAGCTTTTTGCTATTACACATATGGCTTTTCTGCAATCATTTCT 334945

Query: 481 TCCCATTTTGTCTCAAGCCGTTAGTCTTGAAACCACAGGCGGAGTAGAGTTACTTGATGC 540

||||||||||||||||||||||||||||||||||||||||||||||||||||||||||||

Sbjct: 334946 TCCCATTTTGTCTCAAGCCGTTAGTCTTGAAACCACAGGCGGAGTAGAGTTACTTGATGC 335005

Query: 541 GGTATTTTACATGCCTTTTTTCACTGCNNNNNNNNTGAAATACATATTTACACGATTTGC 600

||||||||||||||||||||||||||| |||||||||||||||||||||||||

Sbjct: 335006 GGTATTTTACATGCCTTTTTTCACTGCAAAAAAAATGAAATACATATTTACACGATTTGC 335065

Query: 601 AGGACAGTTTACGATAGTGAGTATGCAGAATAGTTAACACCTTTGTTTTATCCTTTTGTG 660

||||||||||||||||||||||||||||||||||||||||||||||||||||||||||||

Sbjct: 335066 AGGACAGTTTACGATAGTGAGTATGCAGAATAGTTAACACCTTTGTTTTATCCTTTTGTG 335125

Query: 661 TCTTAATTATATGATATAAAGGCGCCTGGCGTTATCGGATAGTAATAGATGCTAGTTATC 720

||||||||||||||||||||||||||||||||||||||||||||||||||||||||||||

Sbjct: 335126 TCTTAATTATATGATATAAAGGCGCCTGGCGTTATCGGATAGTAATAGATGCTAGTTATC 335185

Query: 721 AACATTTCACAATTGAAGGAAATAAAGTTGAAGTACTCAACAAAAACTTACTTCAGAATT 780

||||||||||||||||||||||||||||||||||||||||||||||||||||||||||||

Sbjct: 335186 AACATTTCACAATTGAAGGAAATAAAGTTGAAGTACTCAACAAAAACTTACTTCAGAATT 335245

Query: 781 AAATTTTTGGGGGGAACATAGGCATCCTATGACAGGTGACCACAAGCCCCTCAACGCAAT 840

||||||||||||||||||||||||||||||||||||||||||||||||||||||||||||

Sbjct: 335246 AAATTTTTGGGGGGAACATAGGCATCCTATGACAGGTGACCACAAGCCCCTCAACGCAAT 335305

Query: 841 CTAATATTTTACAAAGTGGTAAAATTCTTTCGTTCTTCGTTTTAATATACAGTCATTTAT 900

||||||||||||||||||||||||||||||||||||||||||||||||||||||||||||

Sbjct: 335306 CTAATATTTTACAAAGTGGTAAAATTCTTTCGTTCTTCGTTTTAATATACAGTCATTTAT 335365

Query: 901 TGATTCTATTACATTAATATTCCTACGCTTCGGCTCACATAATTAACAGGACTTCGAGTC 960

||||||||||||||||||||||||||||||||||||||||||||||||||||||||||||

Sbjct: 335366 TGATTCTATTACATTAATATTCCTACGCTTCGGCTCACATAATTAACAGGACTTCGAGTC 335425

Query: 961 CGTTAAACTTGGGATCAACTAATTTCTACGGAT 993

|||||||||||||||||||||||||||||||||

Sbjct: 335426 CGTTAAACTTGGGATCAACTAATTTCTACGGAT 335458

5’-YORWΔ22

(1,025 basepairs)

Database: W303_MPG_2012_ALAV00000000.fsa

Score = 763.9 bits (5051), Expect = 5.1e-223, P = 5.1e-223
Identities = 1015/1025 (99%), Frame = +1 / +1

Query: 1 ACCCACTGCCCTGAAGTCCAGCAGGTACTGAGGCTTGGATCTCAAAAGCGTGGGGTGCGT 60

||||||||||||||||||||||||||||||||||||||||||||||||||||||||||||

Sbjct: 258966 ACCCACTGCCCTGAAGTCCAGCAGGTACTGAGGCTTGGATCTCAAAAGCGTGGGGTGCGT 259025

Query: 61 GGGCGTGCGCTGCTCGAGGCATTTTAGCCGCTGATAGTGCTGGCTCCAGGGGCCACTGCG 120

||||||||||||||||||||||||||||||||||||||||||||||||||||||||||||

Sbjct: 259026 GGGCGTGCGCTGCTCGAGGCATTTTAGCCGCTGATAGTGCTGGCTCCAGGGGCCACTGCG 259085

Query: 121 CTTGCCATACCCGCACACGGGGTTCTTTGAGGCACTTAATTGACCGGAGATTTAAACGAT 180

||||||||||||||||||||||||||||||||||||||||||||||||||||||||||||

Sbjct: 259086 CTTGCCATACCCGCACACGGGGTTCTTTGAGGCACTTAATTGACCGGAGATTTAAACGAT 259145

Query: 181 ATTGTCGTCGGGGATTAGAAGACATTACTGGTGGGGAACCCTTGATGATAATAGGAAATA 240

||||||||||||||||||||||||||||||||||||||||||||||||||||||||||||

Sbjct: 259146 ATTGTCGTCGGGGATTAGAAGACATTACTGGTGGGGAACCCTTGATGATAATAGGAAATA 259205

Query: 241 TATGCGCAGTATGCTCCATCGTTACGAGATGTTAGCAGCCTCAGAGCATCCTAATGGGAA 300

||||||||||||||||||||| ||||||||||||||||||||||||||||||||||||||

Sbjct: 259206 TATGCGCAGTATGCTCCATCGCTACGAGATGTTAGCAGCCTCAGAGCATCCTAATGGGAA 259265

Query: 301 TATCAATGCATACACACCGGAGCTTGGATATGATAAACGAAATATTCTTGAATCGTGAGA 360

||||||||||||||||||||||||||||||||||||||||||||||||||||||||||||

Sbjct: 259266 TATCAATGCATACACACCGGAGCTTGGATATGATAAACGAAATATTCTTGAATCGTGAGA 259325

Query: 361 TCGCCTGTTTTCAAAACCGTTGGAGGCAGAAACAATTTTGTCACAAGATGGGCATTCTAC 420

||||||||||||||||||||||||||||||||||||||||||||||||||||||||||||

Sbjct: 259326 TCGCCTGTTTTCAAAACCGTTGGAGGCAGAAACAATTTTGTCACAAGATGGGCATTCTAC 259385

Query: 421 CCCATCCTTGCTGTATTATTGTAGTCTCGCTTTCTTTTATGCTGGACAAATGAGACTACT 480

||||||||||||||||||||||||||||||||||||||||||||||||||||||||||||

Sbjct: 259386 CCCATCCTTGCTGTATTATTGTAGTCTCGCTTTCTTTTATGCTGGACAAATGAGACTACT 259445

Query: 481 GCACATTTTTATACGTTCTTGGNNNNNNNNAAAGGTGTGGTTTCGGCATTATCCTGCCGC 540

|||||||||||||||||||||| ||||||||||||||||||||||||||||||

Sbjct: 259446 GCACATTTTTATACGTTCTTGGTTTTTTTTAAAGGTGTGGTTTCGGCATTATCCTGCCGC 259505

Query: 541 ACGTTTCTTGGATAATTCATCCTGATTCTCTATTTTAAACGCTTCAGCCTATCAGGATTT 600

||||||||||||||||||||||||||||||||||||||||||||||||||||||||||||

Sbjct: 259506 ACGTTTCTTGGATAATTCATCCTGATTCTCTATTTTAAACGCTTCAGCCTATCAGGATTT 259565

Query: 601 GGTTTTGATACATACTGCAAGAGTGTATCTCGGGAACAGTCATTTATTCCGCAACAAACT 660

||||||||||||||||||||||||||||||||||||||||||||||||||||||||||||

Sbjct: 259566 GGTTTTGATACATACTGCAAGAGTGTATCTCGGGAACAGTCATTTATTCCGCAACAAACT 259625

Query: 661 TAATTGCGGAACGCGTTAGGCGATTTCTAGCATATATCAAATACCGTTCGCGATTTCTTC 720

||||||||||||||||||||||||||||||||||||||||||||||||||||||||||||

Sbjct: 259626 TAATTGCGGAACGCGTTAGGCGATTTCTAGCATATATCAAATACCGTTCGCGATTTCTTC 259685

Query: 721 TGGGTTCGTCTATTTTCTTTTAAATACTTATTAACGTACTCAAACAACTACACTTCGTTG 780

||||||||||| ||||||||||||||||||||||||||||||||||||||||||||||||

Sbjct: 259686 TGGGTTCGTCTCTTTTCTTTTAAATACTTATTAACGTACTCAAACAACTACACTTCGTTG 259745

Query: 781 TATCTCAGAATGAGATCCCTCAGTATGACAATACATCATTCTAAACGTTCGTAAAACACA 840

||||||||||||||||||||||||||||||||||||||||||||||||||||||||||||

Sbjct: 259746 TATCTCAGAATGAGATCCCTCAGTATGACAATACATCATTCTAAACGTTCGTAAAACACA 259805

Query: 841 TATGAAACAACTTTATAACAAAGCGAACAAAATGGGCAACATGAGATGAAACTCCGCGTC 900

||||||||||||||||||||||||||||||||||||||||||||||||||||||||||||

Sbjct: 259806 TATGAAACAACTTTATAACAAAGCGAACAAAATGGGCAACATGAGATGAAACTCCGCGTC 259865

Query: 901 CCTTAGCTGAACTACCCAAACGTACGAATGCCTGAACAATTAGTTTAGATCCGAGATTCC 960

||||||||||||||||||||||||||||||||||||||||||||||||||||||||||||

Sbjct: 259866 CCTTAGCTGAACTACCCAAACGTACGAATGCCTGAACAATTAGTTTAGATCCGAGATTCC 259925

Query: 961 GCGCTTCCATCATTTAGTATAATCCATATTTTATATAATATATAGGATAAGTAACAGCCC 1020

||||||||||||||||||||||||||||||||||||||||||||||||||||||||||||

Sbjct: 259926 GCGCTTCCATCATTTAGTATAATCCATATTTTATATAATATATAGGATAAGTAACAGCCC 259985

Query: 1021 GCGAA 1025

|||||

Sbjct: 259986 GCGAA 259990

3’-YORWΔ22

(961 basepairs)

Database: W303_MPG_2012_ALAV00000000.fsa

Score = 727.0 bits (4805), Expect = 6.6e-212, P = 6.6e-212
Identities = 961/961 (100%), Frame = +1 / +1

Query: 1 GGACCAACTATCATCCGCTAATTACTGACATTACCAAATGAGATCTGTGAATGGGCAAGA 60

||||||||||||||||||||||||||||||||||||||||||||||||||||||||||||

Sbjct: 260221 GGACCAACTATCATCCGCTAATTACTGACATTACCAAATGAGATCTGTGAATGGGCAAGA 260280

Query: 61 TAAAAAACAAAAATTGAAATGTTTGACGTTATGTAAAACTATTAATTCCTTCGCTTTCGG 120

||||||||||||||||||||||||||||||||||||||||||||||||||||||||||||

Sbjct: 260281 TAAAAAACAAAAATTGAAATGTTTGACGTTATGTAAAACTATTAATTCCTTCGCTTTCGG 260340

Query: 121 CGGTCACAGAATTTGCGTGTAGCTGACTCTTGTTCAATCAATATCATTTGTTACTTTATT 180

||||||||||||||||||||||||||||||||||||||||||||||||||||||||||||

Sbjct: 260341 CGGTCACAGAATTTGCGTGTAGCTGACTCTTGTTCAATCAATATCATTTGTTACTTTATT 260400

Query: 181 TGAAAGTCTGTATTACTGCGCCTATTGTCATCCGTACCAAAGAACGTCAAAAAGAAACAA 240

||||||||||||||||||||||||||||||||||||||||||||||||||||||||||||

Sbjct: 260401 TGAAAGTCTGTATTACTGCGCCTATTGTCATCCGTACCAAAGAACGTCAAAAAGAAACAA 260460

Query: 241 GATAATTTTTGTGCTTACACCATTTATAGATCACTGAGCCCAGAATATCGCTGGAGCTCA 300

||||||||||||||||||||||||||||||||||||||||||||||||||||||||||||

Sbjct: 260461 GATAATTTTTGTGCTTACACCATTTATAGATCACTGAGCCCAGAATATCGCTGGAGCTCA 260520

Query: 301 GTGTAAGTGGCATGAACACAACTCTGACTGATCGCACATATTGCCGTTATCATAAATACT 360

||||||||||||||||||||||||||||||||||||||||||||||||||||||||||||

Sbjct: 260521 GTGTAAGTGGCATGAACACAACTCTGACTGATCGCACATATTGCCGTTATCATAAATACT 260580

Query: 361 AGTTGTACTTGTCAATGCGACGAATGGCATCATGCCTATTATTACGTTCCTCTTTTTCCG 420

||||||||||||||||||||||||||||||||||||||||||||||||||||||||||||

Sbjct: 260581 AGTTGTACTTGTCAATGCGACGAATGGCATCATGCCTATTATTACGTTCCTCTTTTTCCG 260640

Query: 421 TTTCATGTTTCCAGAATGCTATTGAATCTAACACTTCAATTATAAAAAAGAATAAATCCG 480

||||||||||||||||||||||||||||||||||||||||||||||||||||||||||||

Sbjct: 260641 TTTCATGTTTCCAGAATGCTATTGAATCTAACACTTCAATTATAAAAAAGAATAAATCCG 260700

Query: 481 CAATAATTTTAGGCTAATTGTTGTACTGTCAAGCGAACCTAATGGTTAAAATTCAGAGGA 540

||||||||||||||||||||||||||||||||||||||||||||||||||||||||||||

Sbjct: 260701 CAATAATTTTAGGCTAATTGTTGTACTGTCAAGCGAACCTAATGGTTAAAATTCAGAGGA 260760

Query: 541 ACCTTCGACGTAGTCTGATCGCTACTTCTATATCTTATGTTCCCAGTCAATCAAAAGTTG 600

||||||||||||||||||||||||||||||||||||||||||||||||||||||||||||

Sbjct: 260761 ACCTTCGACGTAGTCTGATCGCTACTTCTATATCTTATGTTCCCAGTCAATCAAAAGTTG 260820

Query: 601 ATACTATAATAGCTGCCATTTATACCTGTTAGTTATGGCGATCGTTTATCACGACAAGCC 660

||||||||||||||||||||||||||||||||||||||||||||||||||||||||||||

Sbjct: 260821 ATACTATAATAGCTGCCATTTATACCTGTTAGTTATGGCGATCGTTTATCACGACAAGCC 260880

Query: 661 CAATTTTGGTCGAGATGCAGCCAGTTGCCATCTATTGTTTCCAAAGCATTCGTTTGATTC 720

||||||||||||||||||||||||||||||||||||||||||||||||||||||||||||

Sbjct: 260881 CAATTTTGGTCGAGATGCAGCCAGTTGCCATCTATTGTTTCCAAAGCATTCGTTTGATTC 260940

Query: 721 GTATATGATAGGAAAACAAATTAAACGATGTATCAATTTGCTCATTCATCATAAATTGTG 780

||||||||||||||||||||||||||||||||||||||||||||||||||||||||||||

Sbjct: 260941 GTATATGATAGGAAAACAAATTAAACGATGTATCAATTTGCTCATTCATCATAAATTGTG 261000

Query: 781 TTTGAGGCATTACTAAAAGAGCAGCAGATATCAGTCTGTTGGAATAAAAATCAACTATCA 840

||||||||||||||||||||||||||||||||||||||||||||||||||||||||||||

Sbjct: 261001 TTTGAGGCATTACTAAAAGAGCAGCAGATATCAGTCTGTTGGAATAAAAATCAACTATCA 261060

Query: 841 TCTACTAACTAGTATTTACGTTACTAGTATATTATCATATACGGTGTTAGAAGATGACGC 900

||||||||||||||||||||||||||||||||||||||||||||||||||||||||||||

Sbjct: 261061 TCTACTAACTAGTATTTACGTTACTAGTATATTATCATATACGGTGTTAGAAGATGACGC 261120

Query: 901 AAATGATGAGAAATAGTCATCTAAATTAGTGGAAGCTGAAACGCAAGGATTGATAATGTA 960

||||||||||||||||||||||||||||||||||||||||||||||||||||||||||||

Sbjct: 261121 AAATGATGAGAAATAGTCATCTAAATTAGTGGAAGCTGAAACGCAAGGATTGATAATGTA 261180

Query: 961 A 961

|

Sbjct: 261181 A 261181

*Saccharomyces cerevisiae strain CEN.PK*

5’-YPRCΔ15

(914 baepairs)

Database: CEN.PK2-1Ca_Stanford_2014_JRIV01000000.fsa

Score = 681.2 bits (4500), Expect = 3.6e-198, P = 3.6e-198
Identities = 904/914 (98%), Frame = +1 / +1

Query: 1 CGAGATAGTTTGACGTTCGTTTTTTACTTTGAATATACTCGTAGTCTTTTTACTTTTTGA 60

||||||||||||||||||||||||||||||||||||||||||||||||||||||||||||

Sbjct: 74922 CGAGATAGTTTGACGTTCGTTTTTTACTTTGAATATACTCGTAGTCTTTTTACTTTTTGA 74981

Query: 61 GTATNNNNNNNNNNTGACTAGCAAAATAAAATTAGTAGTCTAAAAAAGAAAGCTCGCACT 120

|||| ||||||||||||||||||||||||||||||||||||||||||||||

Sbjct: 74982 GTATAAAAAAAAAATGACTAGCAAAATAAAATTAGTAGTCTAAAAAAGAAAGCTCGCACT 75041

Query: 121 CAGGATCGAACTAAGGACCAACAGATTTGCAATCTGCTGCGCTACCACTGCGCCATACGA 180

||||||||||||||||||||||||||||||||||||||||||||||||||||||||||||

Sbjct: 75042 CAGGATCGAACTAAGGACCAACAGATTTGCAATCTGCTGCGCTACCACTGCGCCATACGA 75101

Query: 181 GCTTTTGAATTATGGTAATTTTGATTATCCTAGAATGTTATATCTCAATATCTCAATATA 240

||||||||||||||||||||||||||||||||||||||||||||||||||||||||||||

Sbjct: 75102 GCTTTTGAATTATGGTAATTTTGATTATCCTAGAATGTTATATCTCAATATCTCAATATA 75161

Query: 241 TTTTGGACATCTATGAAACACCCATAAAGCAGCCGCTACCAAACAGACAAGATTCAGTAT 300

||||||||||||||||||||||||||||||||||||||||||||||||||||||||||||

Sbjct: 75162 TTTTGGACATCTATGAAACACCCATAAAGCAGCCGCTACCAAACAGACAAGATTCAGTAT 75221

Query: 301 GTAAGGTAAATACCTTTTTGCACAGTTAAACTACCCAAACTTATTAAAGCTTGATAAATT 360

||||||||||||||||||||||||||||||||||||||||||||||||||||||||||||

Sbjct: 75222 GTAAGGTAAATACCTTTTTGCACAGTTAAACTACCCAAACTTATTAAAGCTTGATAAATT 75281

Query: 361 ACTGAAATTCCACCTTTCAGTTAGATTCAGGCCTCATATAGATTAGATATAGGGTACGTA 420

||||||||||||||||||||||||||||||||||||||||||||||||||||||||||||

Sbjct: 75282 ACTGAAATTCCACCTTTCAGTTAGATTCAGGCCTCATATAGATTAGATATAGGGTACGTA 75341

Query: 421 ACATTCTGTCAACCAAGTTGTTGGAATGAAAGTCTAAAATGTCATCTATTCGGTAGCACT 480

||||||||||||||||||||||||||||||||||||||||||||||||||||||||||||

Sbjct: 75342 ACATTCTGTCAACCAAGTTGTTGGAATGAAAGTCTAAAATGTCATCTATTCGGTAGCACT 75401

Query: 481 CATGTTACTAGTATACTGTCACATGCGGTGTAACGTGGGGACATAAAACAGACATCAAAT 540

||||||||||||||||||||||||||||||||||||||||||||||||||||||||||||

Sbjct: 75402 CATGTTACTAGTATACTGTCACATGCGGTGTAACGTGGGGACATAAAACAGACATCAAAT 75461

Query: 541 ATAATGGAAGCTGAAATGCAAAGATCGATAATGTAATAGGAATGAAACATATAAAACGAA 600

||||||||||||||||||||||||||||||||||||||||||||||||||||||||||||

Sbjct: 75462 ATAATGGAAGCTGAAATGCAAAGATCGATAATGTAATAGGAATGAAACATATAAAACGAA 75521

Query: 601 AGGAGAAGTAATGGTAATATTAGTATGTAGAAATACCGATTCAATTTTGGGGATTCTTAT 660

||||||||||||||||||||||||||||||||||||||||||||||||||||||||||||

Sbjct: 75522 AGGAGAAGTAATGGTAATATTAGTATGTAGAAATACCGATTCAATTTTGGGGATTCTTAT 75581

Query: 661 ATTCTCGAGAGAATTTCTAGTATAATCTGTATACATAATATTATAGGCTTTACCAACAAT 720

||||||||||||||||||||||||||||||||||||||||||||||||||||||||||||

Sbjct: 75582 ATTCTCGAGAGAATTTCTAGTATAATCTGTATACATAATATTATAGGCTTTACCAACAAT 75641

Query: 721 GGAATTTCGACAATTATCATATTATTCACCAATTAATCACAAGTTGGTAATGAGTTTGAT 780

||||||||||||||||||||||||||||||||||||||||||||||||||||||||||||

Sbjct: 75642 GGAATTTCGACAATTATCATATTATTCACCAATTAATCACAAGTTGGTAATGAGTTTGAT 75701

Query: 781 AACAAGTTACTTTCTTAACAACGTTAGTATCGTCAAAACACTCGGTTTTACTCGAGCTTG 840

||||||||||||||||||||||||||||||||||||||||||||||||||||||||||||

Sbjct: 75702 AACAAGTTACTTTCTTAACAACGTTAGTATCGTCAAAACACTCGGTTTTACTCGAGCTTG 75761

Query: 841 TAGCACAATAATACCGTGTAGAGTTCTGTATTGTTCTTCTTAGTGCTTGTATATGCTCAT 900

||||||||||||||||||||||||||||||||||||||||||||||||||||||||||||

Sbjct: 75762 TAGCACAATAATACCGTGTAGAGTTCTGTATTGTTCTTCTTAGTGCTTGTATATGCTCAT 75821

Query: 901 CCCGACCTTCCATT 914

||||||||||||||

Sbjct: 75822 CCCGACCTTCCATT 75835

3’-YPRCΔ15

(993 baepairs)

Database: CEN.PK2-1Ca_Stanford_2014_JRIV01000000.fsa

Score = 685.9 bits (4531), Expect = 1.4e-199, P = 1.4e-199
Identities = 931/993 (93%), Frame = +1 / +1

Query: 1 TTTGCGAAACCCTATGCTCTGTTGTTCGGATTTGAAATTTTAAAACTACATTAATGTGTT 60

||||||||||||||||||||||||||||||||||||||||||||||||||||||||||||

Sbjct: 76138 TTTGCGAAACCCTATGCTCTGTTGTTCGGATTTGAAATTTTAAAACTACATTAATGTGTT 76197

Query: 61 AGTTTTTCTTTCTTTCTTTCTTTGTCTTGACGTGATTTGGACTTCTGTCTTGCATTCGCG 120

||||||||||||||||||||||||||||||||||||||||||||||||||||||||||||

Sbjct: 76198 AGTTTTTCTTTCTTTCTTTCTTTGTCTTGACGTGATTTGGACTTCTGTCTTGCATTCGCG 76257

Query: 121 TCCATTCATCTGACCCAATATTCCTTTTGGTTTTGTTATCCTTATAAAAAGAAAGGAAGC 180

||||||||||||||||||||||||||||||||||||||||||||||||||||||||||||

Sbjct: 76258 TCCATTCATCTGACCCAATATTCCTTTTGGTTTTGTTATCCTTATAAAAAGAAAGGAAGC 76317

Query: 181 TTCTTAGAGGGANNNNNNNNNNNNNNNNNNNNNNNNNNNNNNNNNNNNNNNNNNNNNNNN 240

||||||||||||

Sbjct: 76318 TTCTTAGAGGGAAAAAAATGATGAAGAGTAATGCCAAAATATAAATAAATAAATAAATAT 76377

Query: 241 NNNNNNCATTTTCTATTTTTAATAGAATAAGAAGAGCATCTTAAGATTACAATTTCAAGA 300

||||||||||||||||||||||||||||||||||||||||||||||||||||||

Sbjct: 76378 GAAAATCATTTTCTATTTTTAATAGAATAAGAAGAGCATCTTAAGATTACAATTTCAAGA 76437

Query: 301 AATAGTTTACACAGTATATCCAATAACTCCAATAAACTACTTTCCTATACAAATTTCTAT 360

||||||||||||||||||||||||||||||||||||||||||||||||||||||||||||

Sbjct: 76438 AATAGTTTACACAGTATATCCAATAACTCCAATAAACTACTTTCCTATACAAATTTCTAT 76497

Query: 361 GGTGGGATTAATAGTAAAACTTCTGTACTTCTCTAATTCACCAAGAAATTAAGGTAAACA 420

||||||||||||||||||||||||||||||||||||||||||||||||||||||||||||

Sbjct: 76498 GGTGGGATTAATAGTAAAACTTCTGTACTTCTCTAATTCACCAAGAAATTAAGGTAAACA 76557

Query: 421 TCTGGTAAGCACTATCCAGCTTTTTGCTATTACACATATGGCTTTTCTGCAATCATTTCT 480

||||||||||||||||||||||||||||||||||||||||||||||||||||||||||||

Sbjct: 76558 TCTGGTAAGCACTATCCAGCTTTTTGCTATTACACATATGGCTTTTCTGCAATCATTTCT 76617

Query: 481 TCCCATTTTGTCTCAAGCCGTTAGTCTTGAAACCACAGGCGGAGTAGAGTTACTTGATGC 540

||||||||||||||||||||||||||||||||||||||||||||||||||||||||||||

Sbjct: 76618 TCCCATTTTGTCTCAAGCCGTTAGTCTTGAAACCACAGGCGGAGTAGAGTTACTTGATGC 76677

Query: 541 GGTATTTTACATGCCTTTTTTCACTGCNNNNNNNNTGAAATACATATTTACACGATTTGC 600

||||||||||||||||||||||||||| |||||||||||||||||||||||||

Sbjct: 76678 GGTATTTTACATGCCTTTTTTCACTGCAAAAAAAATGAAATACATATTTACACGATTTGC 76737

Query: 601 AGGACAGTTTACGATAGTGAGTATGCAGAATAGTTAACACCTTTGTTTTATCCTTTTGTG 660

||||||||||||||||||||||||||||||||||||||||||||||||||||||||||||

Sbjct: 76738 AGGACAGTTTACGATAGTGAGTATGCAGAATAGTTAACACCTTTGTTTTATCCTTTTGTG 76797

Query: 661 TCTTAATTATATGATATAAAGGCGCCTGGCGTTATCGGATAGTAATAGATGCTAGTTATC 720

||||||||||||||||||||||||||||||||||||||||||||||||||||||||||||

Sbjct: 76798 TCTTAATTATATGATATAAAGGCGCCTGGCGTTATCGGATAGTAATAGATGCTAGTTATC 76857

Query: 721 AACATTTCACAATTGAAGGAAATAAAGTTGAAGTACTCAACAAAAACTTACTTCAGAATT 780

||||||||||||||||||||||||||||||||||||||||||||||||||||||||||||

Sbjct: 76858 AACATTTCACAATTGAAGGAAATAAAGTTGAAGTACTCAACAAAAACTTACTTCAGAATT 76917

Query: 781 AAATTTTTGGGGGGAACATAGGCATCCTATGACAGGTGACCACAAGCCCCTCAACGCAAT 840

||||||||||||||||||||||||||||||||||||||||||||||||||||||||||||

Sbjct: 76918 AAATTTTTGGGGGGAACATAGGCATCCTATGACAGGTGACCACAAGCCCCTCAACGCAAT 76977

Query: 841 CTAATATTTTACAAAGTGGTAAAATTCTTTCGTTCTTCGTTTTAATATACAGTCATTTAT 900

||||||||||||||||||||||||||||||||||||||||||||||||||||||||||||

Sbjct: 76978 CTAATATTTTACAAAGTGGTAAAATTCTTTCGTTCTTCGTTTTAATATACAGTCATTTAT 77037

Query: 901 TGATTCTATTACATTAATATTCCTACGCTTCGGCTCACATAATTAACAGGACTTCGAGTC 960

||||||||||||||||||||||||||||||||||||||||||||||||||||||||||||

Sbjct: 77038 TGATTCTATTACATTAATATTCCTACGCTTCGGCTCACATAATTAACAGGACTTCGAGTC 77097

Query: 961 CGTTAAACTTGGGATCAACTAATTTCTACGGAT 993

|||||||||||||||||||||||||||||||||

Sbjct: 77098 CGTTAAACTTGGGATCAACTAATTTCTACGGAT 77130

5’-YORWΔ22

(1,025 baepairs)

Database: CEN.PK2-1Ca_Stanford_2014_JRIV01000000.fsa

Score = 763.9 bits (5051), Expect = 4.7e-223, P = 4.7e-223

Identities = 1015/1025 (99%), Frame = -1 / +1

Query: 1025 TTCGCGGGCTGTTACTTATCCTATATATTATATAAAATATGGATTATACTAAATGATGGA 966

||||||||||||||||||||||||||||||||||||||||||||||||||||||||||||

Sbjct: 1085 TTCGCGGGCTGTTACTTATCCTATATATTATATAAAATATGGATTATACTAAATGATGGA 1144

Query: 965 AGCGCGGAATCTCGGATCTAAACTAATTGTTCAGGCATTCGTACGTTTGGGTAGTTCAGC 906

||||||||||||||||||||||||||||||||||||||||||||||||||||||||||||

Sbjct: 1145 AGCGCGGAATCTCGGATCTAAACTAATTGTTCAGGCATTCGTACGTTTGGGTAGTTCAGC 1204

Query: 905 TAAGGGACGCGGAGTTTCATCTCATGTTGCCCATTTTGTTCGCTTTGTTATAAAGTTGTT 846

||||||||||||||||||||||||||||||||||||||||||||||||||||||||||||

Sbjct: 1205 TAAGGGACGCGGAGTTTCATCTCATGTTGCCCATTTTGTTCGCTTTGTTATAAAGTTGTT 1264

Query: 845 TCATATGTGTTTTACGAACGTTTAGAATGATGTATTGTCATACTGAGGGATCTCATTCTG 786

||||||||||||||||||||||||||||||||||||||||||||||||||||||||||||

Sbjct: 1265 TCATATGTGTTTTACGAACGTTTAGAATGATGTATTGTCATACTGAGGGATCTCATTCTG 1324

Query: 785 AGATACAACGAAGTGTAGTTGTTTGAGTACGTTAATAAGTATTTAAAAGAAAATAGACGA 726

||||||||||||||||||||||||||||||||||||||||||||||||||||| ||||||

Sbjct: 1325 AGATACAACGAAGTGTAGTTGTTTGAGTACGTTAATAAGTATTTAAAAGAAAAGAGACGA 1384

Query: 725 ACCCAGAAGAAATCGCGAACGGTATTTGATATATGCTAGAAATCGCCTAACGCGTTCCGC 666

||||||||||||||||||||||||||||||||||||||||||||||||||||||||||||

Sbjct: 1385 ACCCAGAAGAAATCGCGAACGGTATTTGATATATGCTAGAAATCGCCTAACGCGTTCCGC 1444

Query: 665 AATTAAGTTTGTTGCGGAATAAATGACTGTTCCCGAGATACACTCTTGCAGTATGTATCA 606

||||||||||||||||||||||||||||||||||||||||||||||||||||||||||||

Sbjct: 1445 AATTAAGTTTGTTGCGGAATAAATGACTGTTCCCGAGATACACTCTTGCAGTATGTATCA 1504

Query: 605 AAACCAAATCCTGATAGGCTGAAGCGTTTAAAATAGAGAATCAGGATGAATTATCCAAGA 546

||||||||||||||||||||||||||||||||||||||||||||||||||||||||||||

Sbjct: 1505 AAACCAAATCCTGATAGGCTGAAGCGTTTAAAATAGAGAATCAGGATGAATTATCCAAGA 1564

Query: 545 AACGTGCGGCAGGATAATGCCGAAACCACACCTTTNNNNNNNNCCAAGAACGTATAAAAA 486

||||||||||||||||||||||||||||||||||| |||||||||||||||||

Sbjct: 1565 AACGTGCGGCAGGATAATGCCGAAACCACACCTTTAAAAAAAACCAAGAACGTATAAAAA 1624

Query: 485 TGTGCAGTAGTCTCATTTGTCCAGCATAAAAGAAAGCGAGACTACAATAATACAGCAAGG 426

||||||||||||||||||||||||||||||||||||||||||||||||||||||||||||

Sbjct: 1625 TGTGCAGTAGTCTCATTTGTCCAGCATAAAAGAAAGCGAGACTACAATAATACAGCAAGG 1684

Query: 425 ATGGGGTAGAATGCCCATCTTGTGACAAAATTGTTTCTGCCTCCAACGGTTTTGAAAACA 366

||||||||||||||||||||||||||||||||||||||||||||||||||||||||||||

Sbjct: 1685 ATGGGGTAGAATGCCCATCTTGTGACAAAATTGTTTCTGCCTCCAACGGTTTTGAAAACA 1744

Query: 365 GGCGATCTCACGATTCAAGAATATTTCGTTTATCATATCCAAGCTCCGGTGTGTATGCAT 306

||||||||||||||||||||||||||||||||||||||||||||||||||||||||||||

Sbjct: 1745 GGCGATCTCACGATTCAAGAATATTTCGTTTATCATATCCAAGCTCCGGTGTGTATGCAT 1804

Query: 305 TGATATTCCCATTAGGATGCTCTGAGGCTGCTAACATCTCGTAACGATGGAGCATACTGC 246

||||||||||||||||||||||||||||||||||||||||||| ||||||||||||||||

Sbjct: 1805 TGATATTCCCATTAGGATGCTCTGAGGCTGCTAACATCTCGTAGCGATGGAGCATACTGC 1864

Query: 245 GCATATATTTCCTATTATCATCAAGGGTTCCCCACCAGTAATGTCTTCTAATCCCCGACG 186

||||||||||||||||||||||||||||||||||||||||||||||||||||||||||||

Sbjct: 1865 GCATATATTTCCTATTATCATCAAGGGTTCCCCACCAGTAATGTCTTCTAATCCCCGACG 1924

Query: 185 ACAATATCGTTTAAATCTCCGGTCAATTAAGTGCCTCAAAGAACCCCGTGTGCGGGTATG 126

||||||||||||||||||||||||||||||||||||||||||||||||||||||||||||

Sbjct: 1925 ACAATATCGTTTAAATCTCCGGTCAATTAAGTGCCTCAAAGAACCCCGTGTGCGGGTATG 1984

Query: 125 GCAAGCGCAGTGGCCCCTGGAGCCAGCACTATCAGCGGCTAAAATGCCTCGAGCAGCGCA 66

||||||||||||||||||||||||||||||||||||||||||||||||||||||||||||

Sbjct: 1985 GCAAGCGCAGTGGCCCCTGGAGCCAGCACTATCAGCGGCTAAAATGCCTCGAGCAGCGCA 2044

Query: 65 CGCCCACGCACCCCACGCTTTTGAGATCCAAGCCTCAGTACCTGCTGGACTTCAGGGCAG 6

||||||||||||||||||||||||||||||||||||||||||||||||||||||||||||

Sbjct: 2045 CGCCCACGCACCCCACGCTTTTGAGATCCAAGCCTCAGTACCTGCTGGACTTCAGGGCAG 2104

Query: 5 TGGGT 1

|||||

Sbjct: 2105 TGGGT 2109

3’-YORWΔ22

(961 baepairs)

Database: CEN.PK2-1Ca_Stanford_2014_JRIV01000000.fsa

Score = 646.7 bits (4270), Expect = 9.0e-188, P = 9.0e-188
Identities = 854/854 (100%), Frame = -1 / +1

Query: 854 TACTAGTTAGTAGATGATAGTTGATTTTTATTCCAACAGACTGATATCTGCTGCTCTTTT 795

||||||||||||||||||||||||||||||||||||||||||||||||||||||||||||

Sbjct: 1 TACTAGTTAGTAGATGATAGTTGATTTTTATTCCAACAGACTGATATCTGCTGCTCTTTT 60

Query: 794 AGTAATGCCTCAAACACAATTTATGATGAATGAGCAAATTGATACATCGTTTAATTTGTT 735

||||||||||||||||||||||||||||||||||||||||||||||||||||||||||||

Sbjct: 61 AGTAATGCCTCAAACACAATTTATGATGAATGAGCAAATTGATACATCGTTTAATTTGTT 120

Query: 734 TTCCTATCATATACGAATCAAACGAATGCTTTGGAAACAATAGATGGCAACTGGCTGCAT 675

||||||||||||||||||||||||||||||||||||||||||||||||||||||||||||

Sbjct: 121 TTCCTATCATATACGAATCAAACGAATGCTTTGGAAACAATAGATGGCAACTGGCTGCAT 180

Query: 674 CTCGACCAAAATTGGGCTTGTCGTGATAAACGATCGCCATAACTAACAGGTATAAATGGC 615

||||||||||||||||||||||||||||||||||||||||||||||||||||||||||||

Sbjct: 181 CTCGACCAAAATTGGGCTTGTCGTGATAAACGATCGCCATAACTAACAGGTATAAATGGC 240

Query: 614 AGCTATTATAGTATCAACTTTTGATTGACTGGGAACATAAGATATAGAAGTAGCGATCAG 555

||||||||||||||||||||||||||||||||||||||||||||||||||||||||||||

Sbjct: 241 AGCTATTATAGTATCAACTTTTGATTGACTGGGAACATAAGATATAGAAGTAGCGATCAG 300

Query: 554 ACTACGTCGAAGGTTCCTCTGAATTTTAACCATTAGGTTCGCTTGACAGTACAACAATTA 495

||||||||||||||||||||||||||||||||||||||||||||||||||||||||||||

Sbjct: 301 ACTACGTCGAAGGTTCCTCTGAATTTTAACCATTAGGTTCGCTTGACAGTACAACAATTA 360

Query: 494 GCCTAAAATTATTGCGGATTTATTCTTTTTTATAATTGAAGTGTTAGATTCAATAGCATT 435

||||||||||||||||||||||||||||||||||||||||||||||||||||||||||||

Sbjct: 361 GCCTAAAATTATTGCGGATTTATTCTTTTTTATAATTGAAGTGTTAGATTCAATAGCATT 420

Query: 434 CTGGAAACATGAAACGGAAAAAGAGGAACGTAATAATAGGCATGATGCCATTCGTCGCAT 375

||||||||||||||||||||||||||||||||||||||||||||||||||||||||||||

Sbjct: 421 CTGGAAACATGAAACGGAAAAAGAGGAACGTAATAATAGGCATGATGCCATTCGTCGCAT 480

Query: 374 TGACAAGTACAACTAGTATTTATGATAACGGCAATATGTGCGATCAGTCAGAGTTGTGTT 315

||||||||||||||||||||||||||||||||||||||||||||||||||||||||||||

Sbjct: 481 TGACAAGTACAACTAGTATTTATGATAACGGCAATATGTGCGATCAGTCAGAGTTGTGTT 540

Query: 314 CATGCCACTTACACTGAGCTCCAGCGATATTCTGGGCTCAGTGATCTATAAATGGTGTAA 255

||||||||||||||||||||||||||||||||||||||||||||||||||||||||||||

Sbjct: 541 CATGCCACTTACACTGAGCTCCAGCGATATTCTGGGCTCAGTGATCTATAAATGGTGTAA 600

Query: 254 GCACAAAAATTATCTTGTTTCTTTTTGACGTTCTTTGGTACGGATGACAATAGGCGCAGT 195

||||||||||||||||||||||||||||||||||||||||||||||||||||||||||||

Sbjct: 601 GCACAAAAATTATCTTGTTTCTTTTTGACGTTCTTTGGTACGGATGACAATAGGCGCAGT 660

Query: 194 AATACAGACTTTCAAATAAAGTAACAAATGATATTGATTGAACAAGAGTCAGCTACACGC 135

||||||||||||||||||||||||||||||||||||||||||||||||||||||||||||

Sbjct: 661 AATACAGACTTTCAAATAAAGTAACAAATGATATTGATTGAACAAGAGTCAGCTACACGC 720

Query: 134 AAATTCTGTGACCGCCGAAAGCGAAGGAATTAATAGTTTTACATAACGTCAAACATTTCA 75

||||||||||||||||||||||||||||||||||||||||||||||||||||||||||||

Sbjct: 721 AAATTCTGTGACCGCCGAAAGCGAAGGAATTAATAGTTTTACATAACGTCAAACATTTCA 780

Query: 74 ATTTTTGTTTTTTATCTTGCCCATTCACAGATCTCATTTGGTAATGTCAGTAATTAGCGG 15

||||||||||||||||||||||||||||||||||||||||||||||||||||||||||||

Sbjct: 781 ATTTTTGTTTTTTATCTTGCCCATTCACAGATCTCATTTGGTAATGTCAGTAATTAGCGG 840

Query: 14 ATGATAGTTGGTCC 1

||||||||||||||

Sbjct: 841 ATGATAGTTGGTCC 854
