# Supplementary figures and images for: Adaptation of the GoldenBraid modular cloning system and creation of a toolkit for the expression of heterologous proteins in yeast mitochondria
Source: BMC Biotechnol. 2017 Nov 13;17:80. doi: 10.1186/s12896-017-0393-y (PMC5683533; doi:10.1186/s12896-017-0393-y)

**Figure S4**

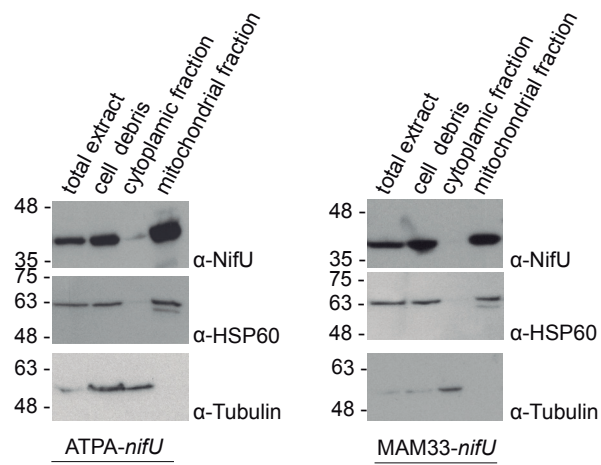

Supplement: Supplementary file 6 — (.tiff) Mitochondrial fractionation from ATPA-NifU and MAM33-NifU cells. Lanes corresponding to total cell extract, pelleted cell debris after centrifugation, cytoplasmic and mitochondria-enriched fractions are shown. Anti-NifU WB is shown in the upper panel, anti-HSP60 WB (mitochondrial matrix marker) is the middle panel and anti-tubulin WB (cytoplasmic marker) is the lower panel. (PDF 677 kb) [file 12896_2017_393_MOESM6_ESM.pdf]
