# Supplementary material for: Adaptation of the GoldenBraid modular cloning system and creation of a toolkit for the expression of heterologous proteins in yeast mitochondria
Source: BMC Biotechnol. 2017 Nov 13;17:80. doi: 10.1186/s12896-017-0393-y (PMC5683533; doi:10.1186/s12896-017-0393-y)
Supplement: Supplementary file 7 — (.doc) Addgene ID numbers of the plasmids described in this work. (DOCX 13 kb) [file 12896_2017_393_MOESM7_ESM.docx]

| **Plasmid** | **Desciption** | **Addgene ID** |
| --- | --- | --- |
| YPRCΔ15α2 | GoldenBraid vector pDGB2α1, adapted for S. cerevisiae genome integration by flanking the GB cassette with recombination arms of homology for integration at the YPRCΔ15 solo LTR loci | 101712 |
| YPRCΔ15α2 | GoldenBraid vector pDGB2α2, adapted for S. cerevisiae genome integration by flanking the GB cassette with recombination arms of homology for integration at the YPRCΔ15 solo LTR loci | 101711 |
| YPRCΔ15Ω1 | GoldenBraid vector pDGB2Ω1, adapted for S. cerevisiae genome integration by flanking the GB cassette with recombination arms of homology for integration at the YPRCΔ15 solo LTR loci | 101710 |
| YPRCΔ15Ω2 | GoldenBraid vector pDGB2Ω2, adapted for S. cerevisiae genome integration by flanking the GB cassette with recombination arms of homology for integration at the YPRCΔ15 solo LTR loci | 101709 |
| YORWΔ22α1 | GoldenBraid vector pDGB2α1, adapted for S. cerevisiae genome integration by flanking the GB cassette with recombination arms of homology for integration at the YORWΔ22 solo LTR loci | 101708 |
| YORWΔ22α2 | GoldenBraid vector pDGB2α2, adapted for S. cerevisiae genome integration by flanking the GB cassette with recombination arms of homology for integration at the YORWΔ22 solo LTR loci | 101707 |
| YORWΔ22Ω1 | GoldenBraid vector pDGB2Ω1, adapted for S. cerevisiae genome integration by flanking the GB cassette with recombination arms of homology for integration at the YORWΔ22 solo LTR loci | 101706 |
| YORWΔ22Ω2 | GoldenBraid vector pDGB2Ω2, adapted for S. cerevisiae genome integration by flanking the GB cassette with recombination arms of homology for integration at the YORWΔ22 solo LTR loci | 101705 |
| pUPD-HygroBR (01-21) | HygroBR in domestication vector | 101704 |
| pUPD-G418R (01-21) | G418R in domestication vector | 101703 |
| pUPD-TDH2t (17-21) | TDH2t in domestication vector | 101702 |
| pUPD-ADH2t (17-21) | ADH2t in domestication vector | 101701 |
| pUPD-Cyc1t (17-21) | Cyc1t in domestication vector | 101700 |
| pUC57-8xHis (13) | 8xHis in domestication vector | 101699 |
| pUPD-MTS2 (12) | MTS2 in domestication vector | 101698 |
| pUPD2-GLRX2MTS (12) | GLRX2MTS in domestication vector | 101697 |
| pUPD2-ATPAMTS (12) | ATPAMTS in domestication vector | 101696 |
| pUPD2-ODPBMTS (12) | ODPBMTS in domestication vector | 101695 |
| pUPD2-ODPAMTS (12) | ODPAMTS in domestication vector | 101694 |
| pUPD-MAM33MTS (12) | MAM33MTS in domestication vector | 101693 |
| pUPD-SOD2MTS (12) | SOD2MTS in domestication vector | 101692 |
| pUPD-Su9MTS (12) | Su9MTS in domestication vector | 101691 |
| pUPD-PGI1 (01-11) | pPGI1 in domestication vector | 101690 |
| pUPD-PGK1 (01-11) | pPGK1 in domestication vector | 101689 |
| pUPD-pPYK1 (01-11) | pPYK1 in domestication vector | 101688 |
| pUPD-pTPI1 (01-11) | pTPI1 in domestication vector | 101687 |
| pUPD-pHXT7 (01-11) | pHXT7 in domestication vector | 101686 |
| pUPD-pTEF2 (01-11) | pTEF2 in domestication vector | 101685 |
| pUPD-pTDH2 (01-11) | pTDH2 in domestication vector | 101684 |
| pUPD-pTDH3 (01-11) | pTDH3 in domestication vector | 101683 |
| pUPD-pGAL1 (01-11) | pGAL1 in domestication vector | 101682 |

**Adaptation of the GoldenBraid modular cloning system and creation of a toolkit for the expression of mitochondrial proteins in yeast.** Ana Pérez-González, Ryan Kniewel, Marcel Veldhuizen, Hemant K. Verma, Mónica Navarro-Rodríguez, Luis M. Rubio and Elena Caro.

**Figure S5**
